# Psychometric properties of the Maternal Postnatal Attachment Scale and the Postpartum Bonding Questionnaire in three German samples

**DOI:** 10.1186/s12884-024-06964-4

**Published:** 2024-11-26

**Authors:** Ariane Göbel, Lisa Lüersen, Eva Asselmann, Petra Arck, Anke Diemert, Susan Garthus-Niegel, Susanne Mudra, Julia Martini

**Affiliations:** 1 Department of Child and Adolescent Psychiatry, Psychotherapy and Psychosomatics, University Medical Center Hamburg-Eppendorf, Hamburg, Germany; 2 Institute for Systems Medicine (ISM), Faculty of Medicine, Medical School Hamburg, Hamburg, Germany; 3 Institute for Mental Health and Behavioral Medicine, Department of Psychology, HMU Health and Medical University, Potsdam, Germany; 4 Division of Experimental Feto-Maternal Medicine, Department of Obstetrics and Fetal Medicine, University Medical Center Hamburg-Eppendorf, Hamburg, Germany; 5 Department of Obstetrics and Fetal Medicine, University Medical Center Hamburg-Eppendorf, Hamburg, Germany; 6 Institute and Policlinic of Occupational and Social Medicine, Faculty of Medicine, Technische Universität Dresden, Dresden, Germany; 7 Department of Childhood and Families, Norwegian Institute of Public Health, Oslo, Norway; 8 Department of Psychiatry and Psychotherapy, Faculty of Medicine, Carl Gustav Carus University Hospital, Technische Universität Dresden, Dresden, Germany

**Keywords:** Mother-infant bonding, Maternal Postnatal Attachment Scale, Postpartum Bonding Questionnaire, Psychometric properties, Factor structure, DREAM study, MARI Study, PAULINE Study, PRINCE study

## Abstract

**Background:**

Forming an emotional bond towards the infant is an important developmental aspect of the mother-child relationship. Two questionnaires frequently used for the assessment of mother-infant bonding, namely the Maternal Postnatal Attachment Scale (MPAS) and the Postpartum Bonding Questionnaire (PBQ), have shown inconclusive psychometric properties. To ensure comparability of results across studies, it is crucial to examine the replicability of psychometric properties and previously proposed factor structures of measurements when adapted to other languages.

**Aim:**

The study aim was to investigate the psychometric properties of the German versions of both MPAS and PBQ, across three different German-speaking study samples.

**Methods:**

Maternal data from three longitudinal studies from Hamburg, Germany (PAULINE-PRINCE study, *N* = 229), and Dresden, Germany (MARI study, *N* = 286; DREAM study, *N* = 1,968), were used to investigate the psychometric properties (descriptive statistics, item difficulty, inter-item correlations) and the factorial structure (confirmatory factor analysis, CFA; principal axis factoring, PAF) of both MPAS and PBQ. Correlations with maternal-fetal bonding, adult romantic attachment style, attachment style to one’s own mother, postpartum depressive symptoms, and education level were investigated.

**Results:**

Across the three samples, both MPAS and PBQ showed convincing results regarding the psychometric properties for their total scores, with satisfying to excellent internal consistencies. A strong correlation between the MPAS and PBQ total scores was observed (*r*=-.71, *p* < .001). In PAF, for both questionnaires, factor structures on subscale level differed across samples and assessment points. For MPAS and PBQ total scores, significant small to medium-sized associations in the expected directions with maternal-fetal bonding and depressive symptoms, as well as for MPAS with adult romantic attachment style, and for PBQ with attachment towards one’s own mother were found. In two samples, higher educated participants reported less optimal MIB.

**Conclusion:**

The results across the three included samples provide evidence for the validity of the construct assessed with the German adaptations of both MPAS and PBQ. However, the factor analytical results on subscale level highlight the need to further investigate the concept of mother-infant bonding in the first year after birth as well as to develop instruments applicable for use in clinical and community samples with satisfying psychometric properties.

**Supplementary Information:**

The online version contains supplementary material available at 10.1186/s12884-024-06964-4.

## Introduction

The development of an emotional bond towards the child is a crucial aspect during the transition to parenthood [1–3]. The subjective parental emotional bond related to the child is an important indicator of the parent-child relationship [4, 5]. Cross-sectional and longitudinal studies revealed small to moderate associations of mother-infant bonding with infant temperament [6] as well as socio-emotional and behavioral development in infancy [7], and early childhood [8, 9]. These results highlight the importance of considering this construct and its interaction with maternal mental health variables and child behavior in research and clinical practice.

In research focusing on the (developing) emotional bond of a parent towards the child, there is a heterogeneity in conceptualization and operationalization of this concept, also leading to a variety of assessment instruments [10]. In this context, the term “parent-child attachment” and “parent-child bonding” have often been used interchangeably [e.g., 2]. However, the term “attachment” is widely associated with Bowlby’s [11] construct definition focusing on the child’s experience of the reciprocal relationship with the caregiver(s) and based on behavior initiated by the child to receive parental care. The parental perspective is in this context also of relevance with focus on ensuring safety and security for the child. To assess this reciprocal parent-child interaction, behavioral observation of the parent-child dyad are most adequate (for an overview of measures, see [12]). As the focus of the current study is on the internal, emotional experience of the mother in the relation with the infant, we thus use the term “mother-infant bonding” (MIB) for concept clarity. So far, divergent definitions of MIB have been proposed. In a recent meta-synthesis of previous concept analytical studies, Nakić Radoš and colleagues [2] defined MIB as a subjective, unidirectional and parent-driven affective experience, manifesting in behavior and involving cognitive (e.g. internal mental representation of the baby) and neurobiological aspects that are potentially important. They further defined MIB as a continuous process starting in the prenatal period with the potential to evolve throughout a child’s and parent’s life. Manifestations of MIB in behavior such as gaze, touch, verbalization, or facial expressions could also be investigated based on observation of the mother-child interaction [13]. Interviews and questionnaires are adequate to assess MIB as the subjective emotional experience in the relationship towards the infant [10]. Especially in research, self-report questionnaires are regularly used to assess MIB.

Measures of MIB also range in their focus from assessing MIB variations in parents from the general population up to severe bonding disorders in clinical samples on the other end of the continuum. Bonding disorders can manifest in form of a lack of experiencing positive feelings towards the infant (as a delayed development, ambivalence, or loss of a previously experienced emotional bond towards the child), feelings of anxiety when being with the infant, feelings of pathological anger (severe form includes physical abuse), or a wish for the infant to disappear temporarily or permanently [14, 15]. In this study, we focus on the dimensional assessment of MIB rather than clinical diagnoses.

Theoretical conceptualizations and empirical results from clinical and community samples indicate a negative association of MIB with mental health variables, especially perinatal depression, and less consistently anxiety and stress [16–18]. Associations with birth experience and (childbirth-related) posttraumatic stress symptoms have been reported, but further research is needed to investigate the relevance of potentially confounding mental health variables (e.g., maternal depression) [19, 20]. These associations might be explained by an interference of MIB by the experienced symptoms [16]. For example, depressive symptoms may include loss of experiencing joy and positive emotions, increased sadness or irritability, a lack of motivation, social withdrawal, ruminating or problems concentrating, as well as feelings of being insufficient as a parent [21]. These symptoms might limit the chance of experiencing positive emotions towards the infant. Postpartum anxiety symptoms might further lead to a rejection of the infant as a form of avoiding the source of current distress on the one hand, but also over-involvement with a strong wish for feeling positive emotions and emotional proximity to the infant on the other hand [15, 16]. Studies highlight the distinctiveness of MIB impairment or disorders from mental health problems, based on clinical observations [14] as well as based on the rather moderate strength of their reported associations in statistical analysis [16]. MIB has further been discussed to be influenced by the experiences with one’s own caregivers [22–24] and representations of attachment in adult close relationships [25, 26]. As proposed by attachment theory, the relationship experiences with own caregivers form internal working models that represent internalized expectations of oneself, the caregivers and relationships (e.g. receiving protection, comfort, encouragement when needed) [27]. These representations have been found to be related to the formation of important and romantic relationships up into adulthood [28]. Contrary to a secure attachment, anxiety in attachment relationships is associated with the fear of losing the important other but also a strong desire for closeness. Avoidance in attachment relationships is associated with the desire to be independent from others and keep own autonomy, going along with feeling uncomfortable with emotional closeness and disclosing feelings [29]. When becoming a parent, the perspective on relationships shifts from the one receiving to the one providing care and security to a dependent person. Bowlby proposed that complementary to the child’s attachment system the caregiving behavioral system develops, comprising emotions, cognitions, and behaviors in the context of the developing parent-child relationship [30, 31]. The parent’s own attachment behavioral system (e.g. needs and strategies) might however interfere with the caregiving behavior system functioning. Insecure attachment has been associated with higher parenting stress [32], maladaptive emotion regulation and coping strategies, as well as less sensitive, consistent, and supportive parenting behavior [33, 34]. Insecurely attached mothers reported feeling less closeness towards the child and greater hostility [34]. Further, studies reported lower MIB in insecurely attached mothers [25, 26, 35, 36], while in other studies no such association was found [37] or the association was mediated by mental health or parenting stress [32, 35, 38]. Regarding the experience of close relationships, social and partner support or relationship satisfaction have been found to be associated with more optimal MIB [17, 39]. Further, among maternal socioeconomic characteristics, education level has been discussed to influence MIB, with mixed results [18]: in case of significant associations [40], higher education was associated with less optimal MIB [41–43]. This association was explained by mothers with higher levels of education being more likely to work and have less time to bond with their child [40] or to experience higher levels of parenting stress, which might influence their emotional experience of the mother-child relationship [36]. Another line of argumentation explains these differences with a reporting bias, with better educated (and potentially older, financially better situated) mothers to report more openly about their experience and therefore also negative emotions in their relationship with the child [41, 43]. Investigating these associations further is relevant for better understanding the development of MIB.

### Assessment of MIB

Associations between MIB and mental health outcomes have been found to be heterogeneous in strength, also partly due to the divergent conceptualizations of the construct and the individual focus of each parent-report instrument developed to assess MIB [16, 44]. Next to interviews (e.g. the Stafford Interview [45]), there is a variety of scales assessing MIB (for a review see Wittkowski et al. [10]). In this study, we focus on two instruments used to asses MIB, namely the Maternal Postnatal Attachment Scale (MPAS) by Condon and Corkindale [5] and the Postpartum Bonding Questionnaire (PBQ) by Brockington [15, 46]. The MPAS and its prenatal counterpart, the Maternal Antenatal Attachment Scale [4], are based on a definition of MIB as the “emotional tie or bond” developing towards the child [5]. The MPAS was developed to assess MIB in a general population. Referring to attachment theory, Condon and Corkindale (1998) describe the underlying feeling of this emotional bond as “love”, which is indicated by the subjective experiences of pleasure in proximity, tolerance, need-gratification, protection of the child, and knowledge acquisition. The scale was developed based on a pool of items derived from unstructured interviews with mothers from Australia within their first year after birth of their child. The factor structure was subsequently tested in a sample of 238 women from the general population in Australia assessed at 4 weeks, 4 months, and 8 months postpartum. Three factors were extracted in each analysis, but with variations in factor loadings depending on the assessment time point. The authors described that a variation in factor structures for the three assessment time points was anticipated, as the parental experience may differ depending on the age of their child. In the currently available version of the MPAS [5] the three dimensions labeled *quality of attachment*, *pleasure in interaction*, and *absence of hostility* are assessed. The total score broadly measures the MIB construct defined by the authors. The psychometric properties of the MPAS have only been investigated in a few studies so far, with acceptable to satisfying internal consistencies for the MPAS total score (α = 0.68 to 0.79) [42, 47] and for the *quality of attachment* subscale (α = 0.57 to 0.76) [42, 47]. Lower values were found for *pleasure in interaction* (α = 0.50 to 0.53) [42, 47] and *absence of hostility* (α = 0.44 to 0.57) [42, 47]. Previous evidence in the international literature regarding the MPAS factor structure has been heterogeneous: factor analytical investigations lead to a six-factor solution for an Italian translation [48] and a two-factor solution after omitting four items in a Portuguese translation [49]. Riera-Martín et al. [50] developed a Spanish adaptation suitable for both mothers and fathers, with three factors comparable to the original questionnaire, after excluding four MPAS items. Dunn et al. [51] could not extract a satisfying factor structure in their British sample, reporting ceiling effects and multiple factor loadings for several items. Even though the MPAS has previously been used in German-speaking samples [52, 53], to our knowledge, its psychometric properties have so far not been investigated in German-speaking mothers.

The PBQ [46] is one of the most widely used MIB measures and shows some conceptual overlap with the MPAS [42]. However, the PBQ was originally developed as a clinical screening instrument for bonding disorders. The PBQ was validated in a British sample including depressed mothers with and without reported MIB disorders and a control group of mothers without reported mental health problems. The extracted factors *impaired bonding* and *rejection and anger* were especially sensitive for identifying women with MIB disorders [46]. The third factor focused on *anxiety about care* for the child. A fourth factor with two items was specifically designed to identify cases at *risk of abuse*. A total score of the scale can be used as a broad indicator when screening for bonding disorders. In a subsequent study, the PBQ was validated in 125 mothers from the UK and New Zealand diagnosed via interview for clinically relevant mother-infant bonding disorders [15]. This study supported the sensitivity of the factors *impaired bonding* to generally identify forms of bonding disorders and *rejection and anger* to identify rejection of the infant. Results for the factors *anxiety about care* and *risk of abuse* were not as convincing. Brockington [15] further pointed out the potential for improvement of the scale. Investigation of the psychometric properties of the PBQ in a German-speaking sample of women with and without diagnosed depression (total *N* = 862) resulted in a one-factor solution with 16 items (PBQ-16) [41]. For adaptations of the PBQ in other languages as well as in clinical and community samples, alternative factor solutions were extracted, ranging from one-factor to four-factor solutions, partly along with substantial item reduction [10, 23, 54–56].

### Aim of this study

The heterogeneous results reported for the MPAS and the PBQ highlight the importance of investigating the factorial structure and construct validity of the questionnaires when translated into another language. This is crucial for cross-cultural comparison of studies and further understanding of the underlying MIB concept and its related constructs. Replication of earlier results further strengthens the confidence in the stability of the construct [57]. Thus, the aim of this study was to investigate and compare the psychometric properties of German translations of the MPAS as well as the original PBQ and PBQ-16 in three German samples.

## Methods

This study is based on maternal data derived from three prospective-longitudinal cohort studies situated in Hamburg (PAULINE-PRINCE study) [58, 59] and Dresden (MARI study [60], DREAM study [61]), Germany.

### PAULINE-PRINCE sample and procedure

The data from mothers living in the area of Hamburg, Germany, were derived from a collaboration between two related, population-based prospective studies situated at the University Medical Center Hamburg-Eppendorf, namely the Prenatal Anxiety and Infant Early Emotional Development (“**P**ränatale **A**ngst **u**nd die emotiona**l**e frühk**in**dliche **E**ntwicklung”, **PAULINE**) study [58] and the **Pr**enatal **I**de**n**tification of **C**hildr**e**n’s Health (**PRINCE**) study [59]. The objective of this collaboration was to investigate prenatal mental health and its relevance for postnatal adjustment, mother-child relationship, and child socio-emotional development. For this aim, a set of psychosocial questionnaires that were used in the PAULINE study were also implemented as a psychosocial module into the PRINCE study design. Pregnant women were included between 09/2014 and 06/2018 and assessed six times across pregnancy up to 24 months postpartum [62, 63]. Women aged ≥ 18 years, pregnant with a singleton child were included in the study. Women with chronic infections, substance abuse, pregnancy after assisted reproductive technologies, or lack of sufficient German language skills, severe pregnancy complications, premature birth (< 37 weeks gestation), and low birth weight (< 2,500 g) were excluded from the study. Interested partners of (expectant) mothers were assessed once prenatally and at 7 months postpartum. All participants signed informed consent forms. The protocols of the PAULINE study and the psychosocial module in PRINCE were approved by the ethics committee of the Hamburg Chamber of Physicians (PV3694 and PV5574). For the current analysis, MPAS data assessed at 7 and 12 months postpartum were pooled from the PAULINE study and the collaboration with the PRINCE study (data sets in the following referred to as MPAS_PP,7M_ and MPAS_PP,12M_). *N* = 218 provided MPAS data at 7 months and *n* = 197 at 12 months postpartum (in total, *N* = 229).

### MARI sample and procedure

The **M**aternal **A**nxiety in **R**elation to **I**nfant Development (**MARI**) study is a prospective-longitudinal regional epidemiological study in 306 (expectant) mothers and their partners from the area of Dresden, Germany (01/2009–09/2012). The study objective was to investigate the role of anxiety and depressive disorders prior to, during, and after pregnancy for perinatal outcomes, maternal health, and offspring development up to 16 months postpartum. Women after their 12th week of gestation, aged < 18 or > 40 years, expecting multiples, or with invasive fertility treatment, severe complications regarding previous pregnancies or infant health, severe physical disease, substance abuse or heroin substitution were not enrolled in the study. More detailed information on the aims, methods, design, and inclusion/ exclusion criteria of the MARI study, including a detailed study flow chart as well as sociodemographic, gynecological, and clinical characteristics of the sample of mothers has been presented elsewhere [60, 64, 65]. Informed consent was obtained from all participants. The study was approved by the Ethics Committee of the Technische Universität Dresden (No: EK 94042007). For the current analysis, PBQ data assessed at 2 months and MPAS data assessed at 4 months postpartum were included (data sets in the following referred to as PBQ_M,2M_ and MPAS_M,4M_). *N* = 281 provided PBQ data at 2 months and *n* = 281 provided MPAS data at four months postpartum (in total *N* = 286).

### DREAM sample and procedure

The Dresden Study on Parenting, Work, and Mental Health (“**DR**esdner Studie zu **E**lternschaft, **A**rbeit und **M**entaler Gesundheit”, **DREAM**) is an ongoing prospective-longitudinal cohort study in (expectant) mothers and their partners in the area of Dresden, Germany (06/2017–ongoing). The study objective is to prospectively examine the association between parental work participation, role distribution, and stress across the perinatal period, including their effects on perinatal outcomes and family (mental) health. Pregnant women and their partners, residing in the Dresden area, with sufficient German skills to complete the study questionnaires were included. To date, seven questionnaire-based assessment waves are being conducted from pregnancy up to 7.5 years postpartum. More detailed information on the aims, methods, design of the DREAM study, including a detailed study flow chart, as well as sample characteristics has been published elsewhere [61]. For the current study, PBQ data assessed at 2 and 14 months postpartum was included. As the data collection of the DREAM study is currently ongoing, the sample size for the assessment point at 14 months postpartum refers to those who had been due at the time of the data extraction (version 10 of the quality-assured data files, released for research in May 2023). Data of participants who did not complete the first PBQ assessment (planned 2 months postpartum) within 6 to 16 weeks postpartum (*n* = 69), and of those who did not complete the second PBQ assessment (planned at 14 months postpartum) within 12 to 16 months postpartum (*n* = 6) [66] were excluded. Further, participants who gave birth to multiples (*n* = 39) were excluded as MPAS and PBQ are not designed for assessing bonding simultaneously to more than one child. To increase comparability between studies regarding potential extreme cases with risk for disrupted bonding [67], those who gave birth to children very preterm (< 32 week of gestation, *n* = 17) or with very low birth weight (< 1,500; *n* = 14) were excluded. *N* = 1,840 provided PBQ data at 2 months and *n* = 1,750 provided PBQ data at 14 months postpartum (in total *N* = 1,968; data sets in the following referred to as PBQ_D,2M_ and PBQ_D,14M_).

### Assessment of MIB

The MPAS [5] consists of 19 items, with different item scoring ranging from two-point to five-point scaling. Items are re-coded prior to analysis for equal weighting, with final item scores ranging from 1 to 5, and a maximum total score of 95. Higher scores indicate more optimal mother-infant bonding. The MPAS was translated into German by the PAULINE-PRINCE and MARI study teams independently of each other for the purpose of these studies, following the recommendations by Bracken and Barona [68]. Prior to the current analysis, the research team of both PAULINE-PRINCE and MARI compared and discussed the two translations for each item as well as their response options to ensure that both translations were comparable in meaning and scoring of each item. The combined German MPAS translation after consent by both study teams can be found in Supplement 1.

The PBQ [46] consists of 25 items, which are scored on a five-point scale, ranging from 0 to 5, with a maximum total score of 125 or 80, respectively. Higher scores indicate more bonding impairment and less optimal mother-infant bonding. The German translation of the PBQ can be accessed via https://marce-gesellschaft.de/materialien/. In addition, a reduced 16 items version of the PBQ (PBQ-16) was proposed by Reck et al. [41].

### Assessment of further variables

To investigate factorial validity of MPAS and PBQ, additional variables were included in this study (for details on assessment time point, study origin, as well as descriptive statistics and scale reliability in the current study for each instrument, see Table 7):

- Maternal-fetal bonding was assessed with a German adaptation of the Maternal Antenatal Attachment Scale [4] with 13 items [69]. Higher scores indicate higher maternal-fetal bonding, with a possible range from 13 to 65.
- Adult romantic attachment style was assessed with the revised version of the Experience in Close Relationships questionnaire (ECR-R; Sibley and Liu 2004) [70] assessing the two dimensions of attachment-related anxiety and avoidance, with mean scale scores ranging from 0 to 7. Higher scores indicate higher attachment-related anxiety and avoidance, respectively.
- Attachment towards one’s own mother was assessed with the Relationship-Specific Attachment Scales (German “Beziehungsspezifische Bindungsskalen für Erwachsene”, BBE) [71]. Based on Bartholomew’s attachment styles in adulthood, two dimensions assess “secure-anxious” (6 items; correlating with Bartholomew’s prototypes “secure” and “fearful”) and “dependent-independent” (8 items; correlating with Bartholomew’s prototype “dismissing”) [71] for the relationship with one’s own mother. The mean scores on the two dimensions can range from 1 (anxious or independent) to 5 (secure or dependent).
- Maternal postpartum depressive symptoms were assessed with the Edinburgh Postnatal Depression Scale (EPDS) [72], a frequently used tool to assess postpartum depression also in German-speaking women [44], with a possible range from 0 to 30, and the 15-item German adaptation of the Center for Epidemiological Studies Depression Scale (“Allgemeine Depressionsskala”, ADS-K) [73], with scores ranging from 0 to 45. In EPDS and ADS-K, higher scores indicate higher levels of depressive symptoms.

### Statistical analysis

Item characteristics, item difficulties (*P*_i_), and inter-item correlations were calculated. *P*_i_ ranges from 0 to 100 and is indicative of the frequencies of high and low scoring in each item. Factor- analytical methods are based on classical test theory. Confirmatory factor analyses (CFA) based on structure equation modeling were conducted to investigate whether the original MPAS, the original PBQ, or the PBQ-16 [41] factor structures were replicated. As the Mardia-Test in lavaan indicated violation of multivariate normality distribution, CFA was run with maximum likelihood estimation with robust standard errors (MLR). As chi square statistics testing for model fit is very sensitive when used in large samples, leading to rejection of the null hypothesis already at smaller mismatch [74], estimation of model fit was based on the following fit indices and the recommendations by Hu & Bentler [75], as well as Schermelleh-Engel et al. [76]: Root Mean Squared Error of Approximation (RMSEA), with values ≤ 0.05 indicating good model fit, and ≤ 0.06 adequate model fit, Standardized Root Mean Square Residual (SRMR), with values ≤ 0.05 indicating good, and ≤ 0.08 adequate model fit, as well as Comparative Fit Index (CFI), with values ≥ 0.95 indicating good model fit. Principal axis factoring (PAF) with oblique (promax) rotation was conducted to investigate the factor loadings without imposing a predefined structure on the data. For each data set, Kaiser-Meyer-Olkin criteria and Bartlett’s test of sphericity confirmed appropriateness for conducting PAF. The number of factors to be extracted were estimated based on investigating the Eigenvalues of each factor (threshold ≥ 1; Kaiser’s criterion), the scree plot, as well as parallel analysis and the optimal coordinate method. Parallel analysis is a technique to retain factors from comparison with simulated data and has been reported to be a more stable estimator for the number of extracted factors compared to solely investigating the Eigenvalues or scree plot [77]. The optimal coordinate method attempts to identify the point of scree by taking gradients of Eigenvalues and preceding coordinates into account [78]. During PAF, factor loadings ≥ 0.30 were considered indicative of importance [79]. Communalities (*h*^2^) are further reported, describing the amount of variance in each item explained by the extracted factors. Scale reliability was assessed with McDonald’s omega (ω), a more sensitive calculation for internal consistency than Cronbach’s alpha [80]. Test-retest reliability of MPAS and PBQ was calculated based on intra-class correlation (ICC). Conducting the factor analytical procedures based on full information maximum likelihood, cases with more than 50% items missing in MPAS or PBQ were excluded. Statistical analyses were conducted using IBM© SPSS 28 [81] and R (v.4.2.1) [82]. Power analyses for conducting a CFA based on structure equation modelling (with the specifications RMSEA = 0.05, α = 0.05 and β = 0.20) estimated a required sample size of *N* = 134 for MPAS (*df* = 143), *N* = 97 (*df* = 255) for the original PBQ, and *N* = 160 (*df* = 105) for PBQ-16. To further investigate MIB construct validity, Pearson correlations of PBQ and MPAS with each other as well as with maternal-fetal bonding were calculated. Associations of MIB with the reported relevant factors adult attachment style in romantic relationships and attachment towards one’s own mother as well as with depressive symptoms were also investigated with Pearson correlations. To examine MIB associations with education level, mothers were divided into dichotomous groups based on their education (1 = not having a university degree vs. 2 = having a university degree). As normality in distribution and homoscedasticity were not given in our analysis, the non-parametric Mann-Whitney-U-Test was used for group comparison.

## Results

### Sample characteristics

Baseline sample characteristics of the three samples are listed in Table 1. In the three studies, a high percentage of participants was well educated, with an average to high household income and only a small percentage reported not being in a relationship. At least half of the participants were expecting their first child at study intake. About half of the participants reported having a girl and most infants in the MARI and DREAM study were born at term.

**Table 1:** Sample characteristics at study intake for the three included studies

Sample characteristics	PAULINE-PRINCE (*N* = 229)			MARI (*N* = 286)				DREAM (*N* = 1,968)		
Maternal age (*M*,* SD*)	33.02	3.54		28.14		4.39		30.19	3.94	
In a relationship^1^ (*N*,* %*)										
Yes	217	94.8		280		97.9		1,936	98.4	
No	6	2.9		6		2.1		21	1.1	
N/A	6	2.9		0		0.0		11	0.6	
Education (*N*,* %*)										
No degree or 9th grade	3	1.2		18		6.3		17	0.9	
10th grade	34	14.9		70		24.5		406	20.6	
High school	53	23.3		105		36.7		426	21.6	
University	132	57.6		93		32.5		1,114	56.6	
N/A	7	3.0		0		0.0		5	0.3	
Parity (*N*, %)										
Primiparous	123	53.7		168		58.7		1,575	80.0	
Multiparous	103	45.0		118		41.2		380	19.3	
N/A	3	1.3		0		0.0		13	0.7	
Infant sex assigned at birth (*N*, %)										
Male	104	45.4		147		51.4		936	47.6	
Female	123	53.7		139		48.6		940	47.8	
N/A	2	0.9		0		0		92	4.7	
Prematurity (*N*, %)	0	0		11		3.8		45	2.3	
Gestational age at birth, in weeks (*M*, *SD*)	39.66	1.04		39.42		1.39		40.29	1.46	
Infant weight at birth, in grams (*M*, *SD*)	3,539.95	409.72		3,441.80		457.41		3,412.28	483.43	
Monthly income, after taxes, in Euro (*N*,* %*)^2^	*Household* income			*Household* income				*Individual* income		
	500–1,000	3	1.3	< 500	20		7.0	≤ 450	61	3.1
	1,001–1,500	6	2.6	500–1,500	99		34.6	451–850	55	2.8
	1,501–2,250	18	7.9	1,500–2,500	89		31.1	851–1,500	452	23.0
	2,251–3,000	19	8.3	2,500–3,500	53		18.5	1,501–2,500	1039	52.8
	3,001–4,000	50	21.8	3,500–4,500	18		6.3	> 2,500	252	12.8
	4,001–5000	66	28.8	> 4,500	7		2.4	N/A	109	5.5
	> 5000	58	25.3	N/A	0		0.0			
	N/A	9	3.9							

*Note. M*, mean; *SD*, standard deviation; ^1^ for MARI assessed at T2 (second trimester of pregnancy); ^2^ for PAULINE-PRINCE and MARI, income of all household members was assessed; for DREAM, individual income for each participant was assessed

### Psychometric properties of MPAS across assessment time points and samples

#### Item characteristics MPAS

For the three data sets MPAS_PP,7M_, MPAS_PP,12M_, and MPAS_M,4M_, the overall high mean scores and *P*_i_ indicate that most participants reported high mother-infant bonding (see Table 2 for details). Across data sets, only item 16 (“time for oneself”) and 17 (“burden of responsibility”) showed higher variance between participants. Overall, low to moderate inter-item correlations were found for all three data sets (see Supplement 2).

**Table 2:** Item characteristics MPAS

		MPAS_PP,7M_ (*n* = 218)					MPAS_PP,12M_ (*n* = 197)					MPAS_M,4M_ (*n* = 281)				
Item		*M*	*SD*	Min	Max	*P*_i_	*M*	*SD*	Min	Max	*P*_i_	*M*	*SD*	Min	Max	*P*_i_
1	Annoyance	4.23	0.71	1.00	5﻿.00	0.85	4.11	0.69	2﻿.00	5﻿.00	0.82	4.54	0.62	3﻿.00	5﻿.00	0.91
2	Difficult child	4.79	0.49	2﻿.00	5﻿.00	0.96	4.67	0.59	2﻿.00	5﻿.00	0.93	4.84	0.43	2﻿.00	5﻿.00	0.97
3	Affection	4.95	0.28	2﻿.00	5﻿.00	0.99	4.97	0.16	4﻿.00	5﻿.00	0.99	4.96	0.29	2﻿.00	5﻿.00	0.99
4	Guilt	4.66	0.70	1﻿.00	5﻿.00	0.93	4.48	0.77	1﻿.00	5﻿.00	0.90	4.87	0.50	1﻿.00	5﻿.00	0.97
5	Competence	4.48	0.76	1﻿.00	5﻿.00	0.90	4.55	0.72	1﻿.00	5﻿.00	0.91	4.86	0.42	3.60	5﻿.00	0.97
6	Tension	4.87	0.41	3.60	5﻿.00	0.97	4.89	0.37	3.60	5﻿.00	0.98	4.87	0.45	1﻿.00	5﻿.00	0.97
7	Pride in baby	4.82	0.51	2.30	5﻿.00	0.96	4.74	0.74	1﻿.00	5﻿.00	0.95	4.67	0.74	1﻿.00	5﻿.00	0.93
8	Prolong/reduce play	4.49	1.34	1﻿.00	5﻿.00	0.90	4.25	1.57	1﻿.00	5﻿.00	0.85	4.77	0.93	1﻿.00	5﻿.00	0.95
9	Separation	3.47	0.99	1﻿.00	5﻿.00	0.69	3.31	0.90	1﻿.00	5﻿.00	0.66	3.69	0.97	1﻿.00	5﻿.00	0.74
10	Enjoyment	4.26	0.76	2.30	5﻿.00	0.85	4.15	0.80	2.30	5﻿.00	0.83	4.38	0.83	1﻿.00	5﻿.00	0.88
11	Thoughts on baby	4.30	0.90	2﻿.00	5﻿.00	0.86	4.17	0.92	2﻿.00	5﻿.00	0.83	3.45	0.98	1﻿.00	5﻿.00	0.69
12	Prolong/reduce time	4.55	1.19	1﻿.00	5﻿.00	0.91	4.56	1.21	1﻿.00	5﻿.00	0.91	4.86	0.74	1﻿.00	5﻿.00	0.97
13	Meet again	4.90	0.34	3﻿.00	5﻿.00	0.98	4.90	0.34	3﻿.00	5﻿.00	0.98	4.79	0.51	2﻿.00	5﻿.00	0.96
14	Own baby	4.96	0.33	1﻿.00	5﻿.00	0.99	4.95	0.32	3﻿.00	5﻿.00	0.99	4.94	0.39	1﻿.00	5﻿.00	0.99
15	Resent baby	4.18	0.90	1﻿.00	5﻿.00	0.84	4.07	0.99	1﻿.00	5﻿.00	0.81	4.60	0.71	1﻿.00	5﻿.00	0.92
16	Time for oneself	3.04	1.07	1﻿.00	5﻿.00	0.61	2.87	1.14	1﻿.00	5﻿.00	0.57	3.49	0.91	1﻿.00	5﻿.00	0.70
17	Burden of responsibility	3.63	1.15	1﻿.00	5﻿.00	0.73	3.60	1.21	1﻿.00	5﻿.00	0.72	4.33	0.90	1﻿.00	5﻿.00	0.87
18	Trust in own judgement	4.75	0.55	2.30	5﻿.00	0.95	4.75	0.59	2.30	5﻿.00	0.95	4.50	0.73	2.30	5﻿.00	0.90
19	Impatience with baby	4.15	0.90	1﻿.00	5﻿.00	0.83	4.03	0.80	2.30	5﻿.00	0.81	4.17	0.87	1﻿.00	5﻿.00	0.83

Note. *M*, mean; *SD*, standard deviation; *P*_i_, item difficulty

#### Confirmatory factor analyses MPAS

CFA based on the original MPAS factor structure [5] with a total scale and the three subscales *quality of attachment*, *pleasure in interaction*, and *absence of hostility* were conducted. Fit indices for MPAS_M,4M_ were closest to be satisfying in comparison to the other two data sets. However, for none of the data sets, fit indices reached the thresholds indicating good model fit for the proposed factor structure (all RMSEA ≥ 0.05, all SRMR ≥ 0.07, all CFI ≤ 0.84; for details see Table 3).

**Table 3:** Fit indices of confirmatory factor analyses for MPAS and PBQ original factor structure as well as the German version PBQ-16

	MPAS original factor structure			PBQ original factor structure			PBQ 16-item factor structure		
	MPAS_PP,7 M_	MPAS_PP,12 M_	MPAS_M,4 M_	PBQ_M,2 M_	PBQ_D,2 M_	PBQ_D,14 M_	PBQ_M,2 M_	PBQ_D,2 M_	PBQ_D,14 M_
χ^2^ (*df*)	381.48 (149)	334.21 (149)	233.92 (149)	942.62 (269)	2,177.65 (269)	1,941.48 (269)	327.17 (104)	1,357.89 (104)	1,090.37 (104)
*p*(Χ^2^)	< 0.01	< 0.01	< 0.01	< 0.01	< 0.01	< 0.01	< 0.01	< 0.01	< 0.01
CFI	0.70	0.74	0.84	0.57	0.76	0.79	0.74	0.80	0.85
RMSEA	0.09	0.08	0.05	0.11	0.09	0.08	0.11	0.11	0.09
*p* RMSEA < 0.05	< 0.01	< 0.01	0.775	< 0.01	< 0.01	< 0.01	< 0.01	< 0.01	< 0.01
SRMR	0.08	0.08	0.07	0.09	0.07	0.06	0.09	0.07	0.06

Note. χ^2^ testing H0 that the model fits the data; CFI, Comparative Fit Index; RMSEA, Root Mean Square Error of Approximation; SRMR, Standardized Root Mean Square Residual

#### Principal axis factoring MPAS

For each MPAS data set, PAF with promax rotation and without a predefined factor structure were conducted for identifying the factor structure fitting the data best (see Supplement 3 for scree plots of each data set).

For MPAS_PP,7M_, the parallel analysis and optimal coordinate method indicated that a four-factor solution fit the data best (Supplement 3). After a PAF restricted to four factors, items 7 (“pride in baby”) and 19 (“impatience with baby”) were excluded due to factor loadings < 0.30. In the final model with 17 items, the four factors explained a total variance of 40% (see Table 4 for details).

**Table 4:** Final pattern matrices for the identified factor structures of MPAS in the three data sets

			MPAS_PP,7M_ (*n* = 218)					MPAS_PP,12M_ (*n* = 197)					MPAS_M,4M_ (*n* = 281)			
Item (original factor)			F2	F3	F1	F4	*h* ^2^	F1	F2	F3	F4	*h* ^2^	F1	F3	F2	*h* ^2^
1	Annoyance	(AH)	(0.31)		0.32		0.50				0.62	0.58	0.50			0.38
2	Difficult child	(AH)	0.45				0.28				0.61	0.35	0.60			0.25
3	Affection	(QA)	0.90				0.68		0.91			0.77	-	-	-	-
4	Guilt	(QA)				0.36	0.30	-	-	-	-	-		0.73		0.48
5	Competence	(QA)				0.44	0.30	0.57				0.36	0.31			0.18
6	Tension	(QA)				0.66	0.41	0.50				0.32	0.53	(0.32)		0.52
7	Pride in baby	(QA)	-	-	-	-	-	-	-	-	-	-	-	-	-	-
8	Play	(PI)		0.44			0.21			0.53		0.26		0.47		0.18
9	Separation	(PI)		0.41			0.19			0.49		0.21			0.72	0.54
10	Enjoyment	(QA)		(0.33)	0.48		0.45			0.42		0.46	0.36			0.24
11	Thoughts on baby	(PI)		0.66			0.41			0.54		0.29			0.50	0.25
12	Prolong/reduce time	(PI)		0.56			0.47			0.49		0.47	-	-	-	-
13	Meet again	(PI)		0.56			0.36		0.37			0.34			0.37	0.25
14	Own Baby	(QA)	0.83				0.55		0.71			0.53	-	-	-	-
15	Resent Baby	(AH)			0.65		0.43	0.51				0.26		0.34	.	0.27
16	Time for oneself	(AH)			0.77		0.49	0.53				0.31	-	-	-	-
17	Burden	(AH)			0.46		0.28	0.51				0.23	0.40			0.31
18	Trust in own judgement	(QA)				0.66	0.32	0.50				0.23	0.54			0.26
19	Impatience	(QA)	-	-	-	-	-				0.43	0.29	0.51			0.24
Eigenvalue			1.93	1.72	1.65	1.42		1.96	1.65	1.39	1.01		1.97	1.16	1.11	
Variance, %			11.4	10.1	9.7	8.3		11.6	9.7	8.2	5.9		14.1	8.3	7.9	
Cumulative variance, %			11.4	21.5	31.2	39.5		11.6	21.6	29.5	35.4		14.1	22.4	30.3	

Note. Only factor loadings ≥ 0.30 listed; In brackets MPAS original factors; QA, quality of attachment; PI, pleasure in interaction; AH, absence of hostility; *h*^2^, communalities

For MPAS_PP,12M_, the parallel analysis and optimal coordinate method indicated that a four-factor solution fit the data best (Supplement 3). A PAF restricted to a two-factor solution was calculated. Item 4 (“guilt”) and 7 (“pride in baby”) had factor loadings < 0.30 and were excluded from further analysis. The final four-factor solution with the remaining 17 items explained 35% of variance (Table 4).

For MPAS_M,4M_, the parallel analysis proposed a four-factor solution and the optimal coordinate method a two-factor solution (Supplement 3). Thus, several factor solutions were investigated. First, a four-factor solution was investigated, with item 7 (“pride in baby”), 14 (“own baby”), and 15 (“resent baby”) having factor loadings < 0.30. As one of the factors of PAF had an Eigenvalue < 1, a three-factor solution was investigated next, with item 7 (“pride in baby”), 3 (“affection”), 12 (“prolong/reduce time with baby”), 14 (“own baby”), and 16 (“time for oneself”) having factor loadings < 0.30. Finally, a two-factor solution was investigated, with factor loadings < 0.30 for item 3 (“affection”), 8 (“prolong/reduce play with baby”), 14 (“own baby”), and factor loadings of 0.31 for item 10 (“enjoyment”), 16 (“time for oneself”), and 19 (“impatience with baby”) each. In comparison, the three-factor solution with 14 items showed higher factor loadings of the items compared to a four- or three-factor solution. With this final three-factor solution, comprising 14 items, 30% of total variance was explained (Table 4).

In sum, divergent MPAS factor solutions were extracted for each data set. Items of the original factors *absence of hostility* and *quality of attachment* loaded on different new factors. The items of the original factor *pleasure in interaction* mostly loaded on one factor in the MPAS_PP,7M_ and MPAS_PP,12M_ data sets, but not in MPAS_M,4M_. Due to the divergent factor solutions, it is not recommended to calculate subscales but use the total score only. Since in all three data sets item 7 (“pride in baby”) had insufficient factor loadings, we eliminated this item from the total score for further analysis in our study.

To investigate how the remaining items would load on one broad factor representing MIB, we repeated PAF for each data set restricted to a 1-factor solution with the remaining 18 items. (Supplement 5). Overall, items loaded sufficiently on one factor across data sets. MPAS item 8 (“playing with the baby”) showed factor loadings between 0.27 and 0.28, slightly below the threshold indicating sufficient factor loadings. As this item showed relevant factor loadings on the factor *pleasure in interaction* (Table 4), we decided to keep this item for further analysis. Item 18 (“I trust my own judgement”) loaded for MPAS_PP,7M_ also slightly below the threshold for sufficient factor loadings (0.27). Item 9 (“separation”) did not load high enough in the data sets MPAS_PP,7M_ (0.19) and MPAS_PP,12M_ (0.22). Item 14 (“own baby”) loaded not sufficiently in MPAS_M,4M_ (0.19). As all of these items showed sufficient factor loadings in some of the three data sets, we decided to also keep them for further analysis.

#### Reliability MPAS

Based on the remaining 18 MPAS items, McDonald’s ω was calculated for MPAS total score in the three data sets. Scale reliability was satisfying to good for MPAS_PP,7M_ (ω = 0.80), MPAS_PP,12M_ (ω = 0.81), and MPAS_M,4M_ (ω = 0.78). For the PAULINE-PRINCE data, MPAS test-retest reliability was good, with ICC = 0.80, 95% CI [0.71, 0.85].

### Psychometric properties of PBQ across assessment time points and samples

#### Item characteristics PBQ

PBQ item characteristics (Table 5) indicated that overall low levels of bonding difficulties were reported. *P*_i_, indicated that most participants scored low on the items in PBQ_M,2M_, as well as for PBQ_D,2M_ and PBQ_D,14M_. The inter-item correlations were overall low to moderate (see Supplement 4 for details on inter-item correlations).

**Table 5:** Item characteristics PBQ

		PBQ_M,2M_ (*n* = 281)					PBQ_D,2M_ (*n* = 1,840)						PBQ_D,14M_ (*n* = 1,750)				
Item		*M*	*SD*	Min	Max	*P*_i_	*M*	*SD*	Min	Max	*P*_i_	*M*		*SD*	Min	Max	*P*_i_
1	Close to baby	0.38	0.63	0.00	3.00	0.13	0.76	0.93	0.00	5.00	0.15	0.68		0.87	0.00	5.00	0.14
2	Wish old days back	0.52	0.77	0.00	5.00	0.10	0.85	0.89	0.00	5.00	0.17	0.91		0.87	0.00	5.00	0.18
3	Distant from baby	0.24	0.61	0.00	5.00	0.05	0.51	0.81	0.00	5.00	0.10	0.44		0.70	0.00	5.00	0.09
4	Love to cuddle	0.31	0.58	0.00	3.00	0.10	0.43	0.75	0.00	5.00	0.09	0.37		0.68	0.00	5.00	0.07
5	Regret having baby	0.06	0.38	0.00	5.00	0.01	0.12	0.46	0.00	5.00	0.02	0.10		0.41	0.00	5.00	0.02
6	Baby not mine	0.10	0.50	0.00	5.00	0.02	0.18	0.56	0.00	5.00	0.04	0.09		0.38	0.00	5.00	0.02
7	Baby winds up	0.65	0.70	0.00	4.00	0.16	0.97	0.91	0.00	5.00	0.19	1.44		0.86	0.00	5.00	0.29
8	Love for baby	0.16	0.49	0.00	3.00	0.05	0.29	0.68	0.00	5.00	0.06	0.26		0.63	0.00	5.00	0.05
9	Feel happy w baby	0.04	0.22	0.00	2.00	0.02	0.06	0.34	0.00	5.00	0.01	0.10		0.42	0.00	5.00	0.02
10	Baby irritates	0.35	0.70	0.00	5.00	0.07	0.78	0.92	0.00	5.00	0.16	0.78		0.89	0.00	5.00	0.16
11	Enjoy playing	0.38	0.63	0.00	3.00	0.13	0.71	0.83	0.00	5.00	0.14	1.03		0.85	0.00	5.00	0.21
12	Baby crying	1.11	0.95	0.00	4.00	0.28	1.29	1.05	0.00	5.00	0.26	1.08		0.99	0.00	5.00	0.22
13	Trapped as mother	0.77	0.97	0.00	5.00	0.15	1.27	1.15	0.00	5.00	0.25	1.21		1.06	0.00	5.00	0.24
14	Angry with baby	0.15	0.40	0.00	2.00	0.07	0.31	0.58	0.00	4.00	0.08	0.65		0.73	0.00	5.00	0.13
15	Resent baby	0.13	0.37	0.00	2.00	0.07	0.29	0.59	0.00	5.00	0.06	0.38		0.66	0.00	5.00	0.08
16	Baby most beautiful	0.40	0.84	0.00	5.00	0.08	0.43	0.80	0.00	5.00	0.09	0.53		0.85	0.00	5.00	0.11
17	Wish baby go away	0.02	0.20	0.00	3.00	0.01	0.05	0.29	0.00	4.00	0.01	0.06		0.34	0.00	5.00	0.01
18	Done harmful things	0.01	0.15	0.00	2.00	0.01	0.02	0.18	0.00	4.00	0.00	0.03		0.22	0.00	3.00	0.01
19	Baby makes anxious	0.39	0.74	0.00	5.00	0.08	0.85	1.03	0.00	5.00	0.17	0.64		0.92	0.00	5.00	0.13
20	Afraid of baby	0.04	0.34	0.00	5.00	0.01	0.08	0.37	0.00	5.00	0.02	0.04		0.28	0.00	5.00	0.01
21	Baby annoys	0.32	0.57	0.00	3.00	0.11	0.55	0.76	0.00	5.00	0.11	0.93		0.81	0.00	4.00	0.23
22	Feeling confident	0.23	0.65	0.00	5.00	0.05	0.35	0.72	0.00	5.00	0.07	0.37		0.82	0.00	5.00	0.07
23	Someone else look after baby	0.04	0.23	0.00	2.00	0.02	0.12	0.43	0.00	4.00	0.03	0.15		0.47	0.00	5.00	0.03
24	Hurting baby	0.01	0.15	0.00	2.00	0.01	0.02	0.18	0.00	5.00	0.00	0.03		0.26	0.00	5.00	0.01
25	Baby easily comforted	1.58	1.08	0.00	5.00	0.32	1.65	1.00	0.00	5.00	0.33	1.44		1.01	0.00	5.00	0.29

Note. *M*, mean; *SD*, standard deviation; *P*_i_, item difficulty

#### Confirmatory factor analyses PBQ

For each PBQ data set, two CFA were calculated. First, for the original four-factor solution with 25 items, and second, for the one-factor solution with 16 items proposed by Reck et al. [41]. For both the original and 16-item solution, fit indices were comparably better when the child was oldest (PBQ_D,14M_). However, none of the factor solutions showed adequate model fit in the three data sets, neither for the PBQ original factor solution (all RMSEA ≥ 0.05, all SRMR ≥ 0.07, all CFI ≤ 0.84) nor for PBQ-16 (all RMSEA ≥ 0.08, all SRMR ≥ 0.06, all CFI ≤ 0.77; see Table 3).

#### Principal axis factoring PBQ

For each PBQ data set, PAF with promax rotation was conducted first with all 25 PBQ items and not imposing a predefined factor structure on the data. See Supplement 4 for scree plots of each data set and Table 6 for the final factor solutions.

**Table 6:** Final pattern matrices for the identified factor structures of PBQ in the three included data sets

			PBQ_M,2M_ (*n*=281)							PBQ_D,2M_ (*n*=1,840)									PBQ_D,14M_ (*n*=1,750)								
Item (original factor)			F5	F1	F2	F3	F4	F6	*h* ^2^	F1	F2		F3		F4		*h* ^2^		F1		F3		F2		F4		*h* ^2^
1	Close to baby	(IB)	0.54						0.55	0.69							0.57				0.40						0.48
2	Wish old days back	(IB)		0.42		(0.38)			0.44				0.33				0.42		-		-		-		-		-
3	Distant from baby	(RA)					0.48		0.46	0.46							0.50						0.30				0.50
4	Love to cuddle	(RA)	0.72						0.46	0.82							0.53				0.60						0.43
5	Regret having baby	(RA)						0.59	0.34				0.68				0.43						0.75				0.59
6	Baby not mine	(IB)	-	-	-	-	-	-	-				0.39				0.28						0.61				0.35
7	Baby winds up	(IB)		0.39					0.56		0.75						0.56		0.81								0.58
8	Love for baby	(IB)	0.72						0.66	0.73							0.62				0.74						0.54
9	Feel happy w baby	(IB)	0.47						0.24	(0.34)			0.44				0.37				0.55						0.37
10	Baby irritates	(IB)						0.85	0.46		0.44						0.36		0.51								0.36
11	Enjoy playing	(RA)	0.84						0.67	0.74							0.50				0.67						0.48
12	Baby crying	(IB)			0.92				0.72						0.74		0.59								0.66		0.54
13	Trapped as mother	(IB)	-	-	-	-	-	-	-	-	-		-		-		-		0.36								0.36
14	Angry with baby	(RA)		0.72					0.50		0.75						0.51		0.76								0.51
15	Resent baby	(IB)		0.58					0.51		0.49						0.47		0.44								0.53
16	Baby most beautiful	(IB)	0.65						0.45	0.68							0.42				0.75						0.47
17	Wish baby go away	(IB)				0.89			0.64				0.92				0.59						0.80				0.53
18	Done harmful things	(AB)	-	-	-	-	-	-	-	-	-		-		-		-		-		-		-		-		-
19	Baby makes anxious	(AC)		0.49					0.27	-	-		-		-		-		-		-		-		-		-
20	Afraid of baby	(AC)		0.61					0.28				0.40				0.18						0.57				0.27
21	Baby annoys	(RA)		0.62					0.58		0.83						0.61		0.87								0.63
22	Feeling confident	(AC)					0.48		0.23	0.38							0.17								0.32		0.16
23	Someone else look after baby	(RA)				0.44			0.25				0.50				0.31						0.45				0.37
24	Hurting baby	(AB)					0.88		0.67	-	-		-		-		-		-		-		-		-		-
25	Baby easily comforted	(AC)			0.71				0.55						0.81		0.63								0.81		0.60
Eigenvalue			2.84	2.30	1.52	1.40	1.29	1.28		3.25	2.41		2.29		1.32				2.72		2.63		2.41		1.36		
Variance, %			12.9	10.5	6.9	6.4	5.9	5.8		15.5	11.5		10.9		6.3				13.0		12.5		11.5		6.7		
Cumulative variance, %			12.9	23.4	30.3	36.7	42.6	48.4		15.5	26.9		37.8		44.1				13.0		25.2		37.0		43.5		

Note. Only factor loadings ≥ 0.30 listed; In brackets PBQ original factors; IB, impaired bonding; RA, rejection and anger; AC, anxiety about care; AB, risk of abuse, *h*^*2*^, communalities

For PBQ_M,2M_, the parallel analysis and optimal coordinate method indicated a six-factor solution to fit the data best. Consequently, a PAF restricted to six factors was conducted. The items 6 (“baby not mine”), 13 (“trapped as mother”), and 18 (“done harmful things to my baby”) did not show sufficient factor loadings and were excluded. The final, reduced six-factor solution explained 48% of variance (Table 6).

For PBQ_D,2M_, the parallel-analysis and optimal coordinate method indicated that a four-factor solution fit the data best. Before conducting a PAF restricted to four factors, the items 18 and 24 were excluded from further analysis within this data set due to their insufficient item characteristics. During PAF, the items 13 (“trapped as mother”) and 19 (“baby makes me anxious”) did not show sufficient factor loadings and were excluded from the final model. The final, reduced four-factor solution explained of 44% of variance (Table 6).

For PBQ_D,14M_, the parallel analysis and optimal coordinate method indicated that a four-factor solution would fit the data best. A PAF restricted to four factors was calculated. The items 2 (“wish old days back”), 18 (“done harmful things to my baby”), 19 (“baby makes me anxious”), and 24 (“feel like hurting baby”) did not load strongly enough on any of the four factors and were excluded from further analysis. The final four-factor solution explained a total of 44% of variance (Table 6).

Overall, divergent factor solutions were extracted for each data set. None of the original factors proposed by Brockington et al. [46] were replicated. Item 18 (“done harmful things to my baby”) did not load strong enough on any of the three data sets and was omitted from the total score for further analysis.

To investigate how the remaining items would load on one broad factor representing MIB, we repeated PAF for each data set restricted to a 1-factor solution with the remaining 24 items. (Supplement 6). Across data sets, most items loaded sufficiently on one factor, with only selective low factor loadings. Item 6 (“baby not mine”) showed low factor loadings in data set PBQ_M,2M_ with 0.18, item 24 (“hurting baby”) only in data set PBQ_M,2M_ and PBQ_D,2M_, with 0.10 and 0.16, respectively. Item 19 (“baby makes me anxious”) loaded slightly below the threshold with 0.28 in data set PBQ_D,14M_. We decided to proceed our analysis using the total score including these items.

#### Reliability PBQ

Based on the remaining 24 PBQ items, scale reliabilities of the PBQ total score were calculated for the three data sets. McDonald’s ω were good for PBQ_M,2M_ (ω = 0.81) and excellent for PBQ_D,2M_ (ω = 0.91) and PBQ_D,14M_ (ω = 0.91). For the DREAM data, PBQ test-retest reliability was moderate to good, with ICC = 0.71, 95% CI [0.72, 0.77].

### Associations of MPAS and PBQ with additional variables

A strong correlation between the 18-item MPAS and 24-item PBQ total scores was observed in the MARI sample (*r* = -.71, *p* < .001), indicating that less optimal MIB assessed with MPAS was associated with higher MIB difficulties assessed with PBQ. Correlations of both instruments with measures for maternal-fetal bonding, adult romantic attachment style, and attachment to one’s own mother, as well as depressive symptoms are reported in Table 7.

**Table 7:** Associations of MPAS and PBQ with further assessed constructs

Construct (Measure, assessment date)	*n*	*M*	*SD*	McDonald’s ω	data set	
PAULINE-PRINCE					MPAS_PP,7 M_	MPAS_PP,12 M_
Maternal-fetal bonding (MAAS, 3. Trimester of pregnancy)	223	55.85	4.22	0.82	*r* = .21**	*r* = .19**
Attachment-related anxiety (ECR-R, 3. Trimester of pregnancy)	222	2.15	0.95	0.91	*r* = − .18*	*r* = − .23**
Attachment-related avoidance (ECR-R, 3. Trimester of pregnancy)	222	1.87	0.75	0.87	*r* = − .39***	*r* = − .23***
Depressive symptoms (EPDS, 7 M pp.)	216	5.33	4.31	0.84	*r* = − .43***	*r* = − .42***
Depressive symptoms (ADS-K, 12 M pp.)	189	7.53	6.37	0.90	*r* = − .38 ***	*r* = − .41***
MARI					PBQ_M,2 M_	MPAS_M,4 M_
Maternal-fetal bonding (MAAS, 2. Trimester of pregnancy)	286	48.28	5.63	0.75	*r* = − .25***	*r* = .40***
Attachment with own mother Secure-anxious dimension (BBE, 4 M pp.)	277	4.13	0.85	0.87	*r* = − .17**	*r = .*13*
Attachment with own mother Dependent-independent dimension (BBE, 4 M pp.)	277	2.92	0.33	0.65	*r* = .05	*r = −* .07
Depressive symptoms (EPDS, 4 M pp.)	283	4.33	3.61	0.83	*r* = .26***	*r* = − .35***
DREAM					PBQ_D,2 M_	PBQ_D,14 M_
Depressive symptoms (EPDS, 2 M pp.)	1,835	5.77	3.87	0.81	*r* = .42 ***	*r* = .23***
Depressive symptoms (EPDS, 14 M pp.)	1,726	5.69	4.23	0.84	*r* = .32***	*r* = .33***

*Note. M*, mean; *SD*, standard deviation; MPAS, Maternal Postnatal Attachment Scale (higher scores indicate more optimal MIB); PBQ, Postpartum Bonding Questionnaire (higher scores indicate MIB difficulties); MAAS, Maternal Antenatal Attachment Scale; ECR-R, Experiences in Close Relationships questionnaire, revised; EPDS, Edinburgh Postnatal Depression Scale; ADS-K, 15-item German adaptation of the Center for Epidemiological Studies Depression Scale (“Allgemeine Depressionsskala”); BBE, Relationship-Specific Attachment Scales (“Beziehungsspezifische Bindungsskalen für Erwachsene”); pp., postpartum; ***p < .001; ** *p* < .01, * *p* < .05

For MIB assessed with the 18-item MPAS total score, small- to medium-sized positive correlations with maternal-fetal bonding were found as well as negative, small- to medium-sized correlations with attachment-related anxiety and avoidance in romantic relationships and depressive symptoms. Further, a small positive association with the secure-anxious attachment dimension to one’s own mother was found. Associations of perceived attachment to one’s own mother on the dependent-independent dimension were not significant. These results indicate that more optimal MIB as assessed with the MPAS was associated with more optimal maternal-fetal bonding, less reported attachment-related anxiety and avoidance in romantic relationships, an attachment to one’s own mother perceived as more secure, as well as less depressive symptoms. Mann-Whitney-U-tests showed that in the PAULINE-PRINCE sample, the group with lower education did not significantly differ in MPAS scores (*M* = 79.34, *SD* = 6.58) from the group with the higher education level at 7 months (*M* = 78.36, *SD* = 6.53), with *U* = 4,741.00, z=-1.322, *p* = .186. At 12 months postpartum, there was also no significant difference between those with lower (*M* = 77.95, *SD* = 7.25) and higher education (*M* = 77.11, *SD* = 6.38), with *U* = 3,825.50, *z*=-1.535, *p* = .125. In the MARI sample, the group with lower education had significantly higher MPAS scores (*M* = 81.67, *SD* = 5.10) than the group with the higher education level (*M* = 79.24, *SD* = 6.27), with *U* = 6,616.00, *z*=-3.251, *p* < .001.

For MIB assessed with the PBQ, a small-to medium-sized, negative association with maternal-fetal bonding was found, as well as a small, negative association with the secure-anxious attachment dimension to one’s own mother. Associations of perceived attachment to one’s own mother on the dependent-independent dimension were not significant. Further, correlations with depressive symptoms were medium-sized, positive, and comparable across different postpartum assessment time points. Mann-Whitney-U-Tests revealed that in the MARI sample, the group with lower education had significantly lower PBQ scores (*M* = 7,59, *SD* = 6,20) than the group with the higher education level (*M* = 10.00, *SD* = 8.10), with *U* = 10,222.000 z = 2.313, *p* = .021. In the DREAM sample at T2, the group with lower education had significantly lower PBQ scores (*M* = 11.32, *SD* = 8.98) than the group with higher education level (*M* = 14.93, *SD* = 10.60), with *U* = 53,4988.50 *z* = 9.084, *p* < .001. At DREAM T3, the group with lower education had significantly lower PBQ scores (*M* = 12.11, *SD* = 9.56) than the group with higher education level (*M* = 15.41, *SD* = 10.57), with *U* = 44,3656.00, *z* = 6.637, *p* = .001. In sum, more bonding difficulties, as indicated by higher PBQ scores, are associated with less optimal maternal-fetal bonding, and as more anxious perceived attachment to one’s own mother, higher depressive symptoms, as well as higher education levels.

## Discussion

The aim of this study was to investigate the psychometric properties of the MPAS and the PBQ in three German samples. In none of the investigated data sets, the originally proposed structure of the MPAS or PBQ (or the German short version PBQ-16) was replicated. Furthermore, divergent factor solutions were identified for each of the included data sets. While some items loaded on the same factor in different samples, others loaded on different factors in each analysis. Our results that the previously proposed factor structures of either MPAS or PBQ were not replicated are in line with research highlighting the heterogeneity in extracted factor solutions for both instruments across different samples and cultural backgrounds [10, 23, 54].

For MPAS, the range of variance in our analysis was rather small and most item characteristics indicated a high level of mother-infant bonding. This is in line with previous research by Dunn et al. [51] pointing out ceiling effects of the MPAS items, which is especially relevant as the MPAS was developed for women in the general population [4]. With limited variance in item scores, relatively small variations within the overall response tendencies of the sample (e.g. caused by subgroup effects) more strongly influence allocation of items on a factor. This might additionally explain the divergent factor solutions for the different samples in our study and also previously reported heterogeneous results in the international literature on the MPAS psychometric properties [48–50].

For the PBQ, the variance in items was also small, with low overall scores and floor effects, indicating low bonding difficulties. For the PBQ, this might be explained by the conceptual focus aiming to identify bonding disorders, including two clinically relevant items probing for child maltreatment [46], which did not differentiate between participants in the current samples.

In both questionnaires there was one item each not loading sufficiently on any factor across samples. For MPAS, we thus recommend treating item 7 (“When I am with the baby and other people are present I feel proud.”) with caution and its characteristics should be investigated thoroughly when using the MPAS in a German sample. Investigating and reporting its item characteristics in future studies is still of relevance to better understand whether this is a consistent result across studies. In both the Spanish [50] and Italian [48] adaptations, this item showed satisfying characteristics, while in the Portuguese adaptation, it’s factor loading was also < 0.30 [49]. One explanation could be that “being proud” in the German language can have a positive connotation (i.e. the emotion of pride), but also a negative connotation (in German “Hochmut”, a form of arrogance; as described for example in van Osch et al., 2013 [83]). This could explain why in the German samples this item was not strongly related to the direct emotional experience in the mother-infant relationship. Qualitative and cross-cultural research can contribute to a better understanding on how such an item is understood in relation to oneself as a parent when answering this question.

For the PBQ, item 18 (“I have done harmful things to my baby”) showed very little variance and insufficient factor loadings. This result is in line with the German adaptation PBQ-16 [41], and some, but not all international factor-analytical studies on the PBQ in clinical and community samples [54]. One explanation might be that harming the child might just be too shameful for the parent to admit, especially in the context of a research survey. This assumption is supported by a study by van Bussel et al. [42], where those participants reporting higher social desirability also reported less bonding difficulties. Further, the parent might fear legal consequences when openly reporting on harmful behavior. Generally, it is of high importance to identify not only parents struggling to develop an emotional bond towards their child, but especially those families with risk for child maltreatment [46], which is why it is relevant to still use this item despite its low factor loadings in our study. Especially in clinical practice, handing out the PBQ with all its 25 items in a supportive, nonjudgmental therapeutic setting helping to reduce social desirable response tendencies can serve as a useful tool to address the different emotional experiences including the risk for harming the child [46].

For both questionnaires, divergent factor solutions were not only found between samples but also across different assessment time points. For the MPAS, the items representing the original factor *pleasure in interaction* showed the highest stability, loading on the same factor when assessed at 7 and 12 months postpartum in the PAULINE-PRINCE sample. At 4 months postpartum in the MARI sample, factor loadings were however not comparable. As Condon & Corkindale [5] pointed out, variation in factor loadings could be expected, as the experience in MIB might differ also depending on the age of the child.

For the PBQ, some items loaded across data sets on the same factor (e.g., item 14 “angry with baby” and 21 “baby annoying”). For the two assessment time points at 2 months postpartum, a comparable set of items loaded on factors labeled F5 in the MARI and F1 in the DREAM sample, respectively. Also in the DREAM sample, F2 at 2 months and F1 at 14 months show similar loadings, as well as F1 at 2 months and F3 at 14 months. Despite some overlapping items, there was no convincing pattern in factor structures. Overall, items did not load across samples on comparable factors. The divergent factor solutions extracted here might also be explained by subgroup differences and are in line with previously reported results on extracted PBQ factors in different studies from the same country, like from Japan [84–86], Portugal [87, 88], and England [89].

For the context of this study, we analyzed associations with further variables based on the total score of each instrument as an overall measure of MIB [5, 15, 46]. An additional PAF restricted to one factor supported the appropriateness of using the total scores, as did satisfying to excellent internal consistency for MPAS and PBQ, respectively. Regarding the conceptualization of MIB, the correlation between MPAS and PBQ total score is comparable to earlier results [42] and demonstrates their conceptual overlap but also a unique perspective that can be assessed with each questionnaire. Both instruments contain items that focus not only on the emotional bond towards the child but also on items tapping into related constructs, such as parenting competence or satisfaction within the maternal role [13]. Especially in the first months postpartum, when an identity of a parent is developing, and parenting self-efficacy is still being formed, these aspects of parenthood might be more strongly interrelated in the early time after birth and develop into more differentiated domains during the first year postpartum [1, 90]. Parents in clinical samples might feel overwhelmed by these changes, as for example less adaptive strategies in coping and emotion regulation or clinical symptoms might interfere with their adaptation process. This could also hinder their development of MIB. As parenting self-efficacy and perception of the maternal role are themselves defined as individual constructs [91–93], influenced by personal, socio-contextual, and child-related factors [94, 95], these experiences might not be directly related to the emotional bond towards the child. More research is needed to clarify which dimensions specifically form the MIB construct, in taking results from previous concept analyses into consideration (e.g [2]). Further, participants reporting stronger maternal-fetal bonding and less attachment-related anxiety or avoidance in adult romantic relationships, as well as a more secure attachment to their own mother reported more optimal MIB to their infant. These associations are in line with previous literature [25, 35, 96] and support the assumption that developing a relationship to the child starts already prenatally and is influenced by one’s own attachment styles [4]. According to attachment theory, the caregiving system, which comprises emotional, cognitive, and behavioral aspects of parenthood, undergoes substantial development during pregnancy and the postpartum period and might be influenced by factors like birth experience, social and partner support, own attachment styles, and experiences with early caregivers [31]. However, associations of the MPAS or PBQ with the dependent-independent dimension of current attachment to one’s own mother were not significant. Internalized working models formed by experiences with own caregiver(s) can influence the development of important relationships up into adulthood, as well as a comfort with closeness, emotion regulation, and coping strategies [33]. However, later experiences with important relationships throughout the live span can influence perceived security in attachment relationships [28]. Investigating their individual relevance for the MIB in multivariate analysis can be an important step to better understand the development of MIB. Across samples, participants reporting more depressive symptoms also reported less optimal bonding to the infant, which is also in line with previous literature [17, 20]. Levels of depressive symptoms were across samples rather low. Still, the reported moderate and partly prospective associations highlight the relevance of mental health variables for the emotional experience and adjustment to parenthood also in samples from the general population. While our analyses focused on bivariate associations, previous research has indicated an interplay between adult attachment styles with maternal mental health variables, like depressive or (child-birth related) posttraumatic stress disorder symptoms, on MIB [22, 26], which further highlights the relevance of investigating the emotional bond related to the child and the general experience of parenthood. As attachment insecurity itself has been proposed to reduce resilience and coping with stressful live events and generally increase vulnerability to mental health problems [30], in clinical samples, these associations might be more pronounced compared to community samples. Socioeconomic factors should also be considered as potential influencing factors. Group comparisons in our analysis showed mixed results: contrary to the PAULINE-PRINCE sample with no significant difference in MPAS scores depending on education, in the samples from Dresden, women with higher education reported lower MIB as reflected by more bonding difficulties. These significant differences are in line with previous literature [41–43]. The nature of this association needs further investigation to identify whether they result from circumstantial (e.g., higher workload and less time with child) or individual aspects (e.g. higher educated women reporting more openly about also negative experiences of MIB). The non-significant difference in the sample from Hamburg might be explained by the comparatively smaller percentage of women with lower education levels compared to the samples from Dresden.

### Strengths and limitations

The most important strength of this study is the investigation and comparison of the psychometric properties of both the MPAS and PBQ in German-speaking samples from three longitudinal studies in Germany, assessed across the first 14 months postpartum. The rare opportunity of directly comparing the results from different data sets deepens the understanding of factorial stability of both instruments across different samples and assessment points. Also, the investigated associations of MPAS and PBQ with each other, with maternal-fetal bonding, adult romantic attachment style, attachment towards one’s own mother, maternal depressive symptoms, and education level helps to better understand the complexity of the MIB construct. Limitations of the current study are that all investigated variables were assessed via self-report only. Even though self-report instruments are generally used to assess especially the emotional experience of MIB, using behavioral measures to assess observable components of MIB (e.g. gaze, touch, facial expression, or vocalization) are of interest to further evaluate the construct and facilitate a comprehensive understanding of MIB. Also, there is still a risk that participants might be reluctant to answer these questions openly. Further, due to the inclusion and exclusion criteria of the individual longitudinal studies and the general risk for a participation bias, comparability between study samples and generalization of our results are limited. Overall, participants were well educated and only a small number was not living in a couple relationship. This might influence our results, given the reported associations between MIB and education. Also, parents living in more demanding or precarious conditions, like as single parent or in poverty, might not have the capacities to participate in a longitudinal study. However, such conditions might influence the developing MIB due to potentially increased stress levels in these households. Finally, as participants with very preterm born infants were excluded from our analysis, their perspective on MIB is not represented in our results.

### Conclusion and implications

The results of this study highlight the need for further investigation of the MIB construct and for further scale development. If used in research, we currently recommend using the total scores of MPAS and PBQ only as an overall indicator of the assessed construct and treat the MPAS item 7 (“pride in baby”) and PBQ item 18 (“done harmful things to my baby”), respectively, with caution due to their insufficient factor loadings in our analysis. This study further indicates that the MPAS and PBQ conceptually overlap but also assess individual MIB facets based on their conceptualization. The reported associations of MPAS with PBQ support the validity of the assessed construct. Our results further support the proposed relation of MIB with maternal-fetal bonding, as well as adult attachment styles related to the relationship with the partner and with one’s own mother. In line with previous work, our factor analytical results highlight the need to develop instruments that assess mother-infant bonding with items showing convincing discriminant validity in samples of mothers from the general population and across different assessment time points. Qualitative study designs could be beneficial for questionnaire development, which might include re-evaluation and rephrasing of existing items, but also development of new items. Questionnaire development should be based on a clear conceptualization of the construct itself, especially in reference to related concepts, as well as on direct maternal report. This research should in addition to the maternal perspective also include the perceived bonding of the father or co-parent towards the child. Longitudinal studies assessing bonding to the child in both parents and beyond infancy can support the understanding of potential change of the construct and its potential dimensions at different stages of parenthood and child development, and further support the distinction of parent-child bonding from related concepts.

## Electronic supplementary material

Below is the link to the electronic supplementary material.

Supplementary Material 1

Supplementary Material 2

Supplementary Material 3

Supplementary Material 4

Supplementary Material 5

Supplementary Material 6

## Data Availability

The data of this study are not openly accessible because after consulting the involved Ethics Committees and due to the sensitive nature of the questions asked in this study, participants were assured that all raw data would remain confidential and not be shared. The data sets analyzed for the current study are available from the corresponding author and PIs of the individual studies on reasonable request.

## Abbreviations

ADS-K: 15-item German adaptation of the Center for Epidemiological Studies Depression Scale (German “Allgemeine Depressionsskala”)
BBE: Relationship-Specific Attachment Scales (German “Beziehungsspezifische Bindungsskalen für Erwachsene”)
CFA: Confirmatory factor analysis
CFI: Comparative Fit Index
DREAM: Dresden study on Parenting, Work, and Mental Health (“**DR**esdner Studie zu **E**lternschaft, **A**rbeit und **M**entaler Gesundheit”)
ECR-R: Experience in Close Relationships Questionnaire, revised
EPDS: Edinburgh Postnatal Depression Scale
ICC: Intra-class correlation
MAAS: Maternal Antenatal Attachment Scale
MARI: **M**aternal **A**nxiety in **R**elation to **I**nfant Development study
MIB: Mother-infant bonding
MPAS: Maternal Postnatal Attachment Scale
PAF: Principal axis factoring
PAULINE: Prenatal Anxiety and Infant Early Emotional Development (“**P**ränatale **A**ngst **u**nd die emotiona**l**e frühk**in**dliche **E**ntwicklung”) study
PRINCE: **Pr**enatal **I**de**n**tification of **C**hildr**e**n’s Health study
PBQ: Postpartum Bonding Questionnaire
RMSEA: Root Mean Squared Error of Approximation
SPSS: Statistical Package for the Social Sciences
SRMR: Standardized Root Mean Square Residual
