# Supplementary material for: Psychometric properties of the Maternal Postnatal Attachment Scale and the Postpartum Bonding Questionnaire in three German samples
Source: BMC Pregnancy Childbirth. 2024 Nov 26;24:789. doi: 10.1186/s12884-024-06964-4 (PMC11590467; doi:10.1186/s12884-024-06964-4)
Supplement: Supplementary file 1 — Supplementary Material 1 [file 12884_2024_6964_MOESM1_ESM.docx]

**Supplement 1
German version Maternal Postnatal Attachment Scale**

Joint version by the study teams of MARI (Maternal Anxiety in Relation to Infant Development; PI J. Martini) and PAULINE (Prenatal Anxiety and Infant Early Emotional Development; PI S. Mudra)

Please cite as: Göbel, A., Li, L.Y., Wittich, J., Hansche, Y., Fischer, J., Martini, J. & Mudra, S. (2024). Mütterliche postnatale Bindungsskala (MPAS): Forschungsversion. Universitätsklinikum Hamburg-Eppendorf, Technische Universität Dresden: Eigendruck

English original Maternal Postnatal Attachment Scale by John T Condon of Flinders University, 2015. Reused under a Creative Commons Attribution 3.0 AU License. File available from: <https://doi.org/10.25957/5dc0f28d14338>

**Diese Fragen beziehen sich auf Ihre Gedanken und Gefühle über Ihr Baby. Bitte kreuzen Sie zur Beantwortung jeder Frage nur ein Kästchen an.**

1. **Wenn ich mich um das Baby kümmere, spüre ich, dass ich ärgerlich oder gereizt werde.**
   - Sehr häufig
   - Häufig
   - Gelegentlich
   - Selten
   - Nie
2. **Wenn ich mich um das Baby kümmere, bekomme ich das Gefühl, dass das Kind absichtlich schwierig ist, oder versucht, mich zu verärgern.**
   - Sehr häufig
   - Häufig
   - Gelegentlich
   - Selten
   - Nie
3. **In den vergangenen zwei Wochen würde ich meine Gefühle für das Baby folgendermaßen beschreiben:**
   - Ablehnung
   - Keine starken Gefühle gegenüber dem Baby
   - Etwas Zuneigung
   - Mäßige Zuneigung
   - Intensive Zuneigung
4. **Im Hinblick auf die gesamte Interaktion/Kommunikation mit dem Baby …**
   - … fühle ich mich sehr schuldig, weil ich nicht mehr beteiligt bin.
   - … fühle ich mich mäßig schuldig, weil ich nicht mehr beteiligt bin.
   - … fühle ich mich etwas schuldig, weil ich nicht mehr beteiligt bin.
   - Ich habe diesbezüglich keine Schuldgefühle.
5. **Wenn ich mich mit dem Baby beschäftige, fühle ich mich:**
   - Sehr inkompetent und ohne Selbstbewusstsein
   - Mäßig inkompetent und ohne Selbstbewusstsein
   - Mäßig kompetent und selbstbewusst
   - Sehr kompetent und selbstbewusst
6. **Wenn ich mit dem Baby zusammen bin, fühle ich mich angespannt und ängstlich.**

- Sehr häufig
- Häufig
- Gelegentlich
- Fast nie

1. **Wenn ich mit dem Baby zusammen bin und andere Menschen auch dabei sind, bin ich stolz auf das Baby.**

- Sehr häufig
- Häufig
- Gelegentlich
- Fast nie

1. **Ich versuche so viel wie ich nur kann, mit dem Baby zu SPIELEN.**

- Das stimmt
- Das stimmt nicht

1. **Wenn ich das Baby verlassen muss, dann…**

- ...fühle ich mich für gewöhnlich eher traurig (oder es fällt mir schwer, das Baby zu verlassen).
- …fühle ich mich oft eher traurig (oder es fällt mir schwer, das Baby zu verlassen).
- …habe ich gemischte Gefühle von Traurigkeit und Erleichterung.
- …fühle ich mich oft eher erleichtert (und es fällt mir leicht zu gehen).
- …fühle ich mich für gewöhnlich eher erleichtert (und es fällt mir leicht zu gehen).

1. **Wenn ich mit dem Baby zusammen bin, erlebe ich:**

- Immer viel Freude und Zufriedenheit
- Häufig viel Freude und Zufriedenheit
- Gelegentlich viel Freude und Zufriedenheit
- Sehr selten viel Freude und Zufriedenheit

1. **Wenn ich nicht mit dem Baby zusammen bin, bemerke ich, dass ich an das Baby denke.**

- Beinah die ganze Zeit
- Sehr häufig
- Häufig
- Gelegentlich
- Gar nicht

1. **Wenn ich mit dem Baby zusammen bin**

- …versuche ich für gewöhnlich die gemeinsame Zeit mit ihr/ihm zu verlängern.
- …versuche ich für gewöhnlich die gemeinsame Zeit mit ihr/ihm so kurz wie möglich zu gestalten.

1. **Wenn ich eine Weile von dem Baby getrennt war, fühle ich bei dem Gedanken, dass ich es gleich wiedersehen werde, für gewöhnlich:**

- Intensive Freude
- Ziemliche Freude
- Etwas Freude
- Nichts
- Negative Gefühle

1. **Gerade, in diesem Moment, sehe ich das Baby**

- Ganz und gar als mein eigenes Baby an
- Ein bisschen als mein eigenes Baby an
- Noch nicht als mein eigenes Baby an

1. **Wenn ich die Dinge denke, die wir wegen des Babys aufgeben mussten, merke ich, dass ich mich:**

- Ziemlich viel darüber ärgere
- Mäßig viel darüber ärgere
- Ein wenig darüber ärgere
- Überhaupt nicht darüber ärgere

1. **In den letzten drei Monaten hatte ich das Gefühl, dass ich nicht genug Zeit für mich selbst und meine eigenen Interessen hatte.**

- Beinah die ganze Zeit
- Sehr häufig
- Gelegentlich
- Gar nicht

1. **Sich um das Baby zu kümmern, ist eine sehr belastende Verantwortung. Ich glaub, diese Aussage stimmt.**

- Ganz genau
- Ziemlich
- Ein wenig
- Überhaupt nicht

1. **Ich vertraue auf mein eigenes Urteil, wenn ich entscheide, was mein Baby braucht.**

- Fast nie
- Gelegentlich
- Die meiste Zeit
- Beinah die ganze Zeit

1. **Wenn ich mit meinem Baby zusammen bin, bin ich für gewöhnlich:**

- Sehr ungeduldig
- Etwas ungeduldig
- Ziemlich geduldig
- Sehr geduldig

**Scoring and Factors:**

Quality of attachment: 3 4 5 6 (7) (10) (14) 18 19

Absence of hostility: 1 2 15 16 17

Pleasure in interaction: all reversed (8 9 11 12 13)

Items in brackets ( ) are reverse scored

To ensure equal weighting of all questions it is recommended that response options be recoded to represent a score of 1 (low attachment) to 5 (high attachment) for every question.

For example:

Question 4 would be scored as: 1; 2.3; 3.6; 5
Question 8 would be (reverse) scored as: 5; 1
