# Supplementary material for: Psychometric properties of the Maternal Postnatal Attachment Scale and the Postpartum Bonding Questionnaire in three German samples
Source: BMC Pregnancy Childbirth. 2024 Nov 26;24:789. doi: 10.1186/s12884-024-06964-4 (PMC11590467; doi:10.1186/s12884-024-06964-4)
Supplement: Supplementary file 2 — Supplementary Material 2 [file 12884_2024_6964_MOESM2_ESM.docx]

| MPAS_PP,7M_ | | | | | | | | | | | | | | | | | | | | |
| --- | --- | --- | --- | --- | --- | --- | --- | --- | --- | --- | --- | --- | --- | --- | --- | --- | --- | --- | --- | --- |
|  | Item 1 | Item 2 | Item 3 | Item 4 | Item 5 | Item 6 | Item 7 | Item 8 | Item 9 | Item 10 | Item 11 | Item 12 | Item 13 | Item 14 | Item 15 | Item 16 | Item 17 | Item 18 | Item 19 |  |
| Item 1 | - | .42^***^ | .44^***^ | .32^***^ | .35^***^ | .31^***^ | .21^**^ | .13 | .13* | .42^***^ | .18^**^ | .31^***^ | .21^***^ | .23^**^ | .31^***^ | .32^***^ | .24^***^ | .20^***^ | .37^***^ |  |
| Item 2 |  | - | .37^***^ | .18^**^ | .21^***^ | .22^***^ | .11 | -.02 | .01 | .16^*^ | .01 | .08 | .10 | .35^***^ | .06 | .12 | .19^**^ | .13 | .21^***^ |  |
| Item 3 |  |  | - | .27^***^ | .24^***^ | .06 | .16^*^ | .03 | -.03 | .19^**^ | .14^*^ | .21^***^ | .19^**^ | .59^***^ | .06 | .17^*^ | .17^*^ | .00 | .19^**^ |  |
| Item 4 |  |  |  | - | .34^***^ | .28^***^ | .17^*^ | .22^***^ | -.04 | .24^***^ | .12 | .16^*^ | .15^*^ | .32^***^ | .14^*^ | .14^*^ | .13 | .22^***^ | .15^*^ |  |
| Item 5 |  |  |  |  | - | .28^***^ | .19^**^ | .02 | -.00 | .23^***^ | .17^*^ | .17^*^ | .14^*^ | .12 | .20^***^ | .12 | .26^***^ | .28^***^ | .14^*^ |  |
| Item 6 |  |  |  |  |  | - | .19^**^ | .12 | .00 | .22^***^ | .05 | .32^***^ | .14^*^ | .06 | .24^***^ | .15^*^ | .18^**^ | .34^***^ | .23^***^ |  |
| Item 7 |  |  |  |  |  |  | - | .05 | .08 | .29^***^ | .18^**^ | .30^***^ | .30^**^ | -.04 | .15^*^ | .16^*^ | .13 | .07 | .13 |  |
| Item 8 |  |  |  |  |  |  |  | - | .10 | .24^***^ | .33^***^ | .21^***^ | .29^***^ | .04 | .20^***^ | -.02 | .01 | .14^*^ | .14^*^ |  |
| Item 9 |  |  |  |  |  |  |  |  | - | .21^***^ | .28^***^ | .28^***^ | .19^***^ | -.12 | .11 | .15^*^ | -.08 | .07 | .10 |  |
| Item 10 |  |  |  |  |  |  |  |  |  | - | .28^***^ | .39^***^ | .25^***^ | .10 | .38^***^ | .43^***^ | .18^**^ | .08 | .30^***^ |  |
| Item 11 |  |  |  |  |  |  |  |  |  |  | - | .31^***^ | .35^***^ | .10 | .08 | .03 | -.07 | .12 | .12 |  |
| Item 12 |  |  |  |  |  |  |  |  |  |  |  | - | .46^***^ | .05 | .32^***^ | .21^***^ | .01 | .08 | .24^***^ |  |
| Item 13 |  |  |  |  |  |  |  |  |  |  |  |  | - | -.03 | .24^***^ | .11 | .07 | .11 | .29^***^ |  |
| Item 14 |  |  |  |  |  |  |  |  |  |  |  |  |  | - | -.06 | .15^*^ | .09 | .09 | .07 |  |
| Item 15 |  |  |  |  |  |  |  |  |  |  |  |  |  |  | - | .41^***^ | .27^***^ | .03 | .23^***^ |  |
| Item 16 |  |  |  |  |  |  |  |  |  |  |  |  |  |  |  | - | .31^***^ | -.02 | .19^**^ |  |
| Item 17 |  |  |  |  |  |  |  |  |  |  |  |  |  |  |  |  | - | .11 | .17^*^ |  |
| Item 18 |  |  |  |  |  |  |  |  |  |  |  |  |  |  |  |  |  | - | .12 |  |
| Item 19 |  |  |  |  |  |  |  |  |  |  |  |  |  |  |  |  |  |  | - |  |

*Note*. ^***^p < .001; ^**^p < .01; ^*^p < .05. (2-tailed); *n* = 218

| MPAS_PP,12M_ | | | | | | | | | | | | | | | | | | | |
| --- | --- | --- | --- | --- | --- | --- | --- | --- | --- | --- | --- | --- | --- | --- | --- | --- | --- | --- | --- |
|  | Item 1 | Item 2 | Item 3 | Item 4 | Item 5 | Item 6 | Item 7 | Item 8 | Item 9 | Item 10 | Item 11 | Item 12 | Item 13 | Item 14 | Item 15 | Item 16 | Item 17 | Item 18 | Item 19 |
| Item 1 | - | .37^***^ | .26^***^ | .24^**^ | .27^***^ | .21^**^ | .09 | .12 | .13 | .36^***^ | .08 | .30^***^ | .14 | .22^**^ | .30^***^ | .27^***^ | .21^**^ | .26^***^ | .40^***^ |
| Item 2 |  | - | .23^**^ | .19^**^ | .12 | .20^**^ | .02 | .03 | .07 | .09 | -.01 | .12 | .05 | .16* | .06 | .11 | .10 | .09 | .25^***^ |
| Item 3 |  |  | - | .15^*^ | .23^**^ | .18^*^ | .10 | .14 | .09 | .26^***^ | .19^**^ | .42^***^ | .46^***^ | .71^***^ | .12 | .12 | .13 | .07 | .05 |
| Item 4 |  |  |  | - | .19^**^ | .25^***^ | .06 | .07 | .03 | .18^*^ | .16^*^ | .23^**^ | .07 | .23^**^ | .14 | .21^**^ | .14^*^ | .10 | .14^*^ |
| Item 5 |  |  |  |  | - | .38^***^ | -.01 | .12 | .05 | .34^***^ | .17^*^ | .18^*^ | .23^**^ | .27^***^ | .23^**^ | .27^***^ | .29^***^ | .36^***^ | .22^**^ |
| Item 6 |  |  |  |  |  | - | -.06 | .01 | .01 | .19^**^ | -.03 | .09 | .14 | .32^**^ | .13 | .34^***^ | .20^**^ | .33^***^ | .12 |
| Item 7 |  |  |  |  |  |  | - | .13 | .07 | .25^***^ | .09 | .10 | .04 | .01 | .23^**^ | .04 | .00 | .16* | -.03 |
| Item 8 |  |  |  |  |  |  |  | - | .23^**^ | .24^***^ | .33^***^ | .33^***^ | .11 | .17^*^ | .03 | .12 | .03 | .01 | -.02 |
| Item 9 |  |  |  |  |  |  |  |  | - | .21^**^ | .25^***^ | .24^**^ | .14^*^ | .05 | .03 | .13 | .05 | -.08 | .07 |
| Item 10 |  |  |  |  |  |  |  |  |  | - | .31^***^ | .38^***^ | .29^***^ | .27^***^ | .39^***^ | .28^***^ | .26^***^ | .21^**^ | .21^**^ |
| Item 11 |  |  |  |  |  |  |  |  |  |  | - | 29^***^ | .20^**^ | .17^*^ | .01 | .11 | -.01 | .12 | .08 |
| Item 12 |  |  |  |  |  |  |  |  |  |  |  | - | .43^***^ | .37^***^ | .25^***^ | .25^***^ | .12 | .12 | .05 |
| Item 13 |  |  |  |  |  |  |  |  |  |  |  |  | - | .33^***^ | .25^***^ | .26^***^ | .20^**^ | .09 | .08 |
| Item 14 |  |  |  |  |  |  |  |  |  |  |  |  |  | - | .16^*^ | .16^*^ | .14 | .08 | .08 |
| Item 15 |  |  |  |  |  |  |  |  |  |  |  |  |  |  | - | .34^***^ | .26^***^ | .21^**^ | .14^*^ |
| Item 16 |  |  |  |  |  |  |  |  |  |  |  |  |  |  |  | - | .31^***^ | .14 | .22^**^ |
| Item 17 |  |  |  |  |  |  |  |  |  |  |  |  |  |  |  |  | - | .19^**^ | .19^**^ |
| Item 18 |  |  |  |  |  |  |  |  |  |  |  |  |  |  |  |  |  | - | .19^**^ |
| Item 19 |  |  |  |  |  |  |  |  |  |  |  |  |  |  |  |  |  |  | - |
| *Note. ***p* < .001; ***p* < .01; **p* < .05. (2-tailed); *n* = 197 | | | | | | | | | | | | | | | | | | | |

| MPAS_M,4M_ | | | | | | | | | | | | | | | | | | | |  |
| --- | --- | --- | --- | --- | --- | --- | --- | --- | --- | --- | --- | --- | --- | --- | --- | --- | --- | --- | --- | --- |
|  | Item 1 | Item 2 | Item 3 | Item 4 | Item 5 | Item 6 | Item 7 | Item 8 | Item 9 | Item 10 | Item 11 | Item 12 | Item 13 | Item 14 | Item 15 | Item 16 | Item 17 | Item 18 | Item 19 | |
| Item 1 | 1 | .23^***^ | .09 | .24^***^ | 22^***^ | .36^***^ | .22^***^ | .12 | .14^*^ | .36^***^ | ,04 | .14^*^ | .30^**^ | .03 | .36^***^ | .32^***^ | .36^***^ | .21^***^ | .31^***^ | |
| Item 2 |  | 1 | .15^*^ | .05 | .10 | .26^***^ | .18^**^ | -.05 | .05 | .19^**^ | -.01 | .16^**^ | .16^**^ | .08 | .14^*^ | .17^**^ | .26^***^ | .24^***^ | .22^***^ | |
| Item 3 |  |  | 1 | .16^**^ | -.01 | .15^*^ | .19^**^ | .12^*^ | .09 | .07 | .04 | .17^**^ | .21^**^ | .14^*^ | -.01 | .03 | .13^*^ | .10 | .15^*^ | |
| Item 4 |  |  |  | 1 | .21^***^ | .39^***^ | .14^*^ | .28^***^ | .08 | .17^**^ | .07 | .21^***^ | .15^*^ | .04 | .33^***^ | .10 | .28^***^ | .16^**^ | .08 | |
| Item 5 |  |  |  |  | 1 | .35^***^ | .14^*^ | .18^**^ | -.01 | .21^***^ | -.02 | .06 | .07 | -.01 | .10 | .18^**^ | .19^**^ | .20^**^ | .23^***^ | |
| Item 6 |  |  |  |  |  | 1 | .16^**^ | .12^*^ | -.01 | .20^**^ | -.08 | .19^**^ | .17^**^ | .05 | .26^***^ | .22^***^ | .32^***^ | .37^***^ | .23^***^ | |
| Item 7 |  |  |  |  |  |  | 1 | .12^*^ | .16^**^ | .33^***^ | .14^*^ | .17^**^ | .27^***^ | .10 | .15^*^ | .15^*^ | .19^**^ | .21^***^ | .13^*^ | |
| Item 8 |  |  |  |  |  |  |  | 1 | .10 | .13^*^ | .13^*^ | .20^**^ | .05 | .16^**^ | .16^**^ | .21^***^ | .15^*^ | .04 | .06 | |
| Item 9 |  |  |  |  |  |  |  |  | 1 | .23^***^ | .34^***^ | .14^*^ | .34^***^ | .07 | .17^**^ | .20^**^ | .18^**^ | .13^*^ | .20^**^ | |
| Item 10 |  |  |  |  |  |  |  |  |  | 1 | .10 | .18^**^ | .22^***^ | .11 | .25^***^ | .29^***^ | .25^***^ | .18^**^ | .22^***^ | |
| Item 11 |  |  |  |  |  |  |  |  |  |  | 1 | .13^*^ | .19^**^ | -.03 | .10 | .11 | .03 | -.03 | .11 | |
| Item 12 |  |  |  |  |  |  |  |  |  |  |  | 1 | .34^***^ | .05 | .04 | .06 | .26^***^ | .02 | .09 | |
| Item 13 |  |  |  |  |  |  |  |  |  |  |  |  | 1 | .11 | .22^**^ | .14^*^ | .21^**^ | .10 | .15^*^ | |
| Item 14 |  |  |  |  |  |  |  |  |  |  |  |  |  | 1 | .03 | .02 | .02 | .06 | .11 | |
| Item 15 |  |  |  |  |  |  |  |  |  |  |  |  |  |  | 1 | .31^***^ | .34^***^ | .20^**^ | .11 | |
| Item 16 |  |  |  |  |  |  |  |  |  |  |  |  |  |  |  | 1 | .31^***^ | ,11 | .13^*^ | |
| Item 17 |  |  |  |  |  |  |  |  |  |  |  |  |  |  |  |  | 1 | .24^**^ | .19^**^ | |
| Item 18 |  |  |  |  |  |  |  |  |  |  |  |  |  |  |  |  |  | 1 | .28^***^ | |
| Item 19 |  |  |  |  |  |  |  |  |  |  |  |  |  |  |  |  |  |  | 1 | |
| *Note*. ^***^p < .001; ^**^p < .01; ^*^p < .05. (2-tailed), *n* = 288 | | | | | | | | | | | | | | | | | | | | |
