# Supplementary material for: Psychometric properties of the Maternal Postnatal Attachment Scale and the Postpartum Bonding Questionnaire in three German samples
Source: BMC Pregnancy Childbirth. 2024 Nov 26;24:789. doi: 10.1186/s12884-024-06964-4 (PMC11590467; doi:10.1186/s12884-024-06964-4)
Supplement: Supplementary file 3 — Supplementary Material 3 [file 12884_2024_6964_MOESM3_ESM.docx]

**Supplement 3**

*Inter-item correlations PBQ data sets*

| PBQ_M,2M_ | | | | | | | | | | | | | | | | | | | | | | | | | |  |  |
| --- | --- | --- | --- | --- | --- | --- | --- | --- | --- | --- | --- | --- | --- | --- | --- | --- | --- | --- | --- | --- | --- | --- | --- | --- | --- | --- | --- |
|  | Item 1 | PBQ 2 | PBQ 3 | PBQ 4 | PBQ 5 | PBQ 6 | PBQ 7 | PBQ 8 | PBQ 9 | PBQ 10 | PBQ 11 | PBQ 12 | PBQ 13 | PBQ 14 | PBQ 15 | PBQ 16 | PBQ 17 | PBQ 18 | PBQ 19 | PBQ 20 | PBQ 21 | PBQ 22 | PBQ 23 | PBQ 24 | PBQ 25 | |  |
| Item 1 | - | .35^***^ | .48^***^ | .41^***^ | .23^**^ | .10 | .37^***^ | .28^***^ | .25^***^ | .54^***^ | .52^***^ | .18^**^ | .34^***^ | .26^***^ | .34^***^ | .44^***^ | .32^***^ | .10 | .12 | .07 | .37^***^ | .08 | .17^***^ | .02 | .21^**^ | |  |
| Item 2 |  | - | .27^***^ | .15^*^ | .19^**^ | -.01 | .38^***^ | .21^***^ | .10 | .27^***^ | .21^***^ | .14^*^ | .51^***^ | .32^***^ | .42^***^ | .16^**^ | .36^***^ | .16^**^ | .31^***^ | .34^***^ | .40^***^ | .08 | .25^***^ | .09 | .22^***^ | |  |
| Item 3 |  |  | - | .26^***^ | .19^**^ | .13^*^ | .27^***^ | .21^***^ | .19^**^ | .36^***^ | .35^***^ | .21^***^ | .24^***^ | .19^**^ | .32^***^ | .22^***^ | .14^*^ | .12^*^ | .14^*^ | .11 | .18^**^ | .28^***^ | .19^**^ | .36^***^ | .21^***^ | |  |
| Item 4 |  |  |  | - | .09 | .11 | .23^***^ | .14^*^ | .32^***^ | .41^***^ | .61^***^ | .22^***^ | .13^*^ | .13^*^ | .18^**^ | .39^***^ | .05 | .07 | .09 | .14^*^ | .27^***^ | .18^**^ | .04 | -.05 | .17^**^ | |  |
| Item 5 |  |  |  |  | - | .02 | .07 | .44^***^ | .10 | .13^*^ | .16^**^ | .10 | .41^***^ | .10 | .19^**^ | .33^***^ | .13^*^ | .24^***^ | .10 | .01 | .19^**^ | .03 | .22^***^ | -.02 | .09 | |  |
| Item 6 |  |  |  |  |  | - | .13^*^ | -.00 | .03 | .22^***^ | .19^**^ | .02 | .17^***^ | .12^*^ | .08 | -.00 | -.02 | -.02 | .10 | .17^**^ | .11 | .02 | .03 | .03 | .08 | |  |
| Item 7 |  |  |  |  |  |  | - | .36^***^ | .12^*^ | .27^***^ | .26^***^ | .35^***^ | .31^***^ | .45^***^ | .40^***^ | .20^**^ | .15^*^ | .15^**^ | .26^***^ | .08 | .64^***^ | .14^*^ | .13^*^ | .15^**^ | .37^***^ | |  |
| Item 8 |  |  |  |  |  |  |  | - | .11 | .16^**^ | .20^**^ | .13^*^ | .33^***^ | .20^**^ | .29^***^ | .26^***^ | .06 | .02 | .30^***^ | .10 | .35^***^ | .11 | .18^**^ | -.05 | .11 | |  |
| Item 9 |  |  |  |  |  |  |  |  | - | .33^***^ | .37^***^ | .03 | .11 | .26^***^ | .24^***^ | .30^***^ | .07 | .20^***^ | .12^*^ | .13^*^ | .23^***^ | .11 | -.03 | -.02 | .14^*^ | |  |
| Item 10 |  |  |  |  |  |  |  |  |  | - | .56^***^ | .09 | .27^***^ | .24^***^ | .35^***^ | .50^***^ | .30^***^ | .07 | .10 | .09 | .23^***^ | .07 | .04 | -.03 | .16^**^ | |  |
| Item 11 |  |  |  |  |  |  |  |  |  |  | - | .12 | .24^***^ | .31^***^ | .35^***^ | .49^**^ | .09 | .10 | .10 | .17^**^ | .29^***^ | .13^*^ | .12^*^ | -.02 | .17^**^ | |  |
| Item 12 |  |  |  |  |  |  |  |  |  |  |  | - | .25^***^ | .18^**^ | .12^*^ | .07 | -.01 | .04 | .18^**^ | .10 | .28^***^ | .07 | .10 | .09 | .63^***^ | |  |
| Item 13 |  |  |  |  |  |  |  |  |  |  |  |  | - | .36^***^ | .43^***^ | .27^***^ | .27^***^ | .20^**^ | .27^***^ | .27^***^ | .37^***^ | .02 | .25^***^ | -.00 | .27^***^ | |  |
| Item 14 |  |  |  |  |  |  |  |  |  |  |  |  |  | - | .50^***^ | .22^***^ | .06 | .21^***^ | .23^***^ | .36^***^ | .55^***^ | .06 | .21^***^ | .15^*^ | .20^**^ | |  |
| Item 15 |  |  |  |  |  |  |  |  |  |  |  |  |  |  | - | .25^***^ | .26^***^ | .23^***^ | .28^***^ | .37^***^ | .43^***^ | .08 | .24^***^ | .03 | .18^**^ | |  |
| Item 16 |  |  |  |  |  |  |  |  |  |  |  |  |  |  |  | - | .20^**^ | .07 | .05 | .03 | .22^**^ | .12^*^ | .03 | -.05 | .11 | |  |
| Item 17 |  |  |  |  |  |  |  |  |  |  |  |  |  |  |  |  | - | -.01 | .00 | .04 | .14^*^ | -.00 | .38^***^ | -.01 | .02 | |  |
| Item 18 |  |  |  |  |  |  |  |  |  |  |  |  |  |  |  |  |  | - | .11 | -.01 | .25^***^ | -.03 | -.02 | -.01 | .15^*^ | |  |
| Item 19 |  |  |  |  |  |  |  |  |  |  |  |  |  |  |  |  |  |  | - | .35^***^ | .37^***^ | .04 | .10 | -.02 | .24^***^ | |  |
| Item 20 |  |  |  |  |  |  |  |  |  |  |  |  |  |  |  |  |  |  |  | - | .26^***^ | .03 | .08 | -.01 | .20^***^ | |  |
| Item 21 |  |  |  |  |  |  |  |  |  |  |  |  |  |  |  |  |  |  |  |  | - | .02 | .18^***^ | .03 | .31^***^ | |  |
| Item 22 |  |  |  |  |  |  |  |  |  |  |  |  |  |  |  |  |  |  |  |  |  | - | -.01 | .34^***^ | .16^**^ | |  |
| Item 23 |  |  |  |  |  |  |  |  |  |  |  |  |  |  |  |  |  |  |  |  |  |  | - | -.02 | .07 | |  |
| Item 24 |  |  |  |  |  |  |  |  |  |  |  |  |  |  |  |  |  |  |  |  |  |  |  | - | .18^**^ | |  |
| Item 25 |  |  |  |  |  |  |  |  |  |  |  |  |  |  |  |  |  |  |  |  |  |  |  |  | - | |  |
| *Note*. ^***^p < .01; ^**^p < .01; ^*^p < .05. (2-tailed), *n*= 288 | | | | | | | | | | | | | | | | | | | | | | | | | | | |

| PBQ_D,2M_ | | | | | | | | | | | | | | | | | | | | | | | | | |  |  |
| --- | --- | --- | --- | --- | --- | --- | --- | --- | --- | --- | --- | --- | --- | --- | --- | --- | --- | --- | --- | --- | --- | --- | --- | --- | --- | --- | --- |
|  | Item 1 | PBQ 2 | PBQ 3 | PBQ 4 | PBQ 5 | PBQ 6 | PBQ 7 | PBQ 8 | PBQ 9 | PBQ 10 | PBQ 11 | PBQ 12 | PBQ 13 | PBQ 14 | PBQ 15 | PBQ 16 | PBQ 17 | PBQ 18 | PBQ 19 | PBQ 20 | PBQ 21 | PBQ 22 | PBQ 23 | PBQ 24 | PBQ 25 | |  |
| Item 1 | - | .45*** | .65*** | .52*** | .35*** | .36*** | .38*** | .59*** | .30*** | .36*** | .49*** | .27*** | .41*** | .35*** | .37*** | .43*** | .34*** | .05* | .19*** | .23*** | .35*** | .33*** | .31*** | .10*** | .31*** | |  |
| Item 2 |  | - | .46*** | .34*** | .48*** | .29*** | .42*** | .39*** | .26*** | .35*** | .37*** | .32*** | .51*** | .35*** | .42*** | .32*** | .39*** | .01 | .20*** | .23*** | .40*** | .20*** | .33*** | .03 | .31*** | |  |
| Item 3 |  |  | - | .46*** | .39*** | .39*** | .37*** | .51*** | .30*** | .39*** | .41*** | .26*** | .39*** | .35*** | .41*** | .38*** | .37*** | .04 | .24*** | .26*** | .37*** | .27*** | .39*** | .08*** | .23*** | |  |
| Item 4 |  |  |  | - | .29*** | .27*** | .29*** | .55*** | .42*** | .29*** | .57*** | .21*** | .29*** | .28*** | .36*** | .45*** | .31*** | .01 | .13*** | .19*** | .30*** | .29*** | .27*** | .08*** | .22*** | |  |
| Item 5 |  |  |  |  | - | .30*** | .27*** | .43*** | .35*** | .27*** | .29*** | .21*** | .34*** | .24*** | .38*** | .30*** | .53*** | -.00 | .11*** | .23*** | .30*** | .17*** | .34*** | .07** | .21*** | |  |
| Item 6 |  |  |  |  |  | - | .23*** | .37*** | .29*** | .24*** | .31*** | .15*** | .26*** | .16*** | .34*** | .26*** | .36*** | .04 | .19*** | .28*** | .25*** | .19*** | .29*** | .06* | .15*** | |  |
| Item 7 |  |  |  |  |  |  | - | .31*** | .21*** | .46*** | .31*** | .37*** | .38*** | .52*** | .45*** | .28*** | .26*** | -.01 | .19*** | .19*** | .59*** | .14*** | .24*** | .10*** | .35*** | |  |
| Item 8 |  |  |  |  |  |  |  | - | .48*** | .29*** | .51*** | .19*** | .32*** | .29*** | .40*** | .57*** | .42*** | .03 | .11*** | .24*** | .34*** | .23*** | .29*** | .13*** | .21*** | |  |
| Item 9 |  |  |  |  |  |  |  |  | - | .20*** | .36*** | .13*** | .20*** | .15*** | .30*** | .40*** | .46*** | .04 | .09*** | .26*** | .18*** | .17*** | .28*** | .09*** | .12*** | |  |
| Item 10 |  |  |  |  |  |  |  |  |  | - | .31*** | .32*** | .36*** | .40*** | .41*** | .24*** | .25*** | .04 | .33*** | .25*** | .41*** | .22*** | .25*** | .17*** | .29*** | |  |
| Item 11 |  |  |  |  |  |  |  |  |  |  | - | .14*** | .32*** | .27*** | .40*** | .47*** | .31*** | .02 | .16*** | .19*** | .34*** | .27*** | .28*** | .07** | .19*** | |  |
| Item 12 |  |  |  |  |  |  |  |  |  |  |  | - | .33*** | .36*** | .25*** | .16*** | .18*** | .01 | .18*** | .15*** | .35*** | .15*** | .20*** | .07** | .62*** | |  |
| Item 13 |  |  |  |  |  |  |  |  |  |  |  |  | - | .35*** | .38*** | .27*** | .28*** | .05* | .26*** | .22** | .38*** | .17*** | .29*** | .08*** | .30*** | |  |
| Item 14 |  |  |  |  |  |  |  |  |  |  |  |  |  | - | .43*** | .19*** | .25*** | .00 | .15*** | .21*** | .58*** | .16*** | .24*** | .13*** | .32*** | |  |
| Item 15 |  |  |  |  |  |  |  |  |  |  |  |  |  |  | - | .40*** | .39*** | -.01 | .16*** | .23*** | .49*** | .16*** | .34*** | .11*** | .24*** | |  |
| Item 16 |  |  |  |  |  |  |  |  |  |  |  |  |  |  |  | - | .28*** | .05* | .08** | .14*** | .32*** | .23*** | .26*** | .09*** | .20*** | |  |
| Item 17 |  |  |  |  |  |  |  |  |  |  |  |  |  |  |  |  | - | -.02 | .11*** | .28*** | .30*** | .13*** | .42*** | .11*** | .15*** | |  |
| Item 18 |  |  |  |  |  |  |  |  |  |  |  |  |  |  |  |  |  | - | .09*** | .00 | -.01 | .09*** | .07** | -.01 | .02 | |  |
| Item 19 |  |  |  |  |  |  |  |  |  |  |  |  |  |  |  |  |  |  | - | .28*** | .23*** | .19*** | .17*** | .03 | .15*** | |  |
| Item 20 |  |  |  |  |  |  |  |  |  |  |  |  |  |  |  |  |  |  |  | - | .22*** | .17*** | .26*** | .09*** | .13*** | |  |
| Item 21 |  |  |  |  |  |  |  |  |  |  |  |  |  |  |  |  |  |  |  |  | - | .13*** | .29*** | .10*** | .34*** | |  |
| Item 22 |  |  |  |  |  |  |  |  |  |  |  |  |  |  |  |  |  |  |  |  |  | - | .26*** | .06** | .23*** | |  |
| Item 23 |  |  |  |  |  |  |  |  |  |  |  |  |  |  |  |  |  |  |  |  |  |  | - | .09*** | .23*** | |  |
| Item 24 |  |  |  |  |  |  |  |  |  |  |  |  |  |  |  |  |  |  |  |  |  |  |  | - | .07** | |  |
| Item 25 |  |  |  |  |  |  |  |  |  |  |  |  |  |  |  |  |  |  |  |  |  |  |  |  | - | |  |
| *Note*. ^***^p < .01; ^**^p < .01; ^*^p < .05. (2-tailed); *n* = 1,840 | | | | | | | | | | | | | | | | | | | | | | | | | | | |

| PBQ_D,14M_ | | | | | | | | | | | | | | | | | | | | | | | | | |  |  |
| --- | --- | --- | --- | --- | --- | --- | --- | --- | --- | --- | --- | --- | --- | --- | --- | --- | --- | --- | --- | --- | --- | --- | --- | --- | --- | --- | --- |
|  | Item 1 | PBQ 2 | PBQ 3 | PBQ 4 | PBQ 5 | PBQ 6 | PBQ 7 | PBQ 8 | PBQ 9 | PBQ 10 | PBQ 11 | PBQ 12 | PBQ 13 | PBQ 14 | PBQ 15 | PBQ 16 | PBQ 17 | PBQ 18 | PBQ 19 | PBQ 20 | PBQ 21 | PBQ 22 | PBQ 23 | PBQ 24 | PBQ 25 | |  |
| Item 1 | - | .38*** | .61*** | .51*** | .39*** | .36*** | .40*** | .46*** | .30*** | .36*** | .43*** | .38*** | .38** | .37*** | .40*** | .42*** | .30*** | .12*** | .16*** | .24*** | .41*** | .23*** | .35*** | .22*** | .37*** | |  |
| Item 2 |  | - | .41*** | .29*** | .47*** | .28*** | .36*** | .33*** | .25*** | .28*** | .40*** | .31*** | .49*** | .30*** | .40*** | .30*** | .33*** | .08*** | .17*** | .19*** | .32*** | .15*** | .29*** | .13*** | .25*** | |  |
| Item 3 |  |  | - | .45*** | .46*** | .42*** | .41*** | .42*** | .34*** | .41*** | .42*** | .33*** | .41*** | .40*** | .49*** | .39*** | .39*** | .18*** | .22*** | .30*** | .44*** | .19*** | .42*** | .21*** | .31*** | |  |
| Item 4 |  |  |  | - | .34*** | .27*** | .33*** | .47*** | .37*** | .28*** | .44*** | .27*** | .29*** | .31*** | .38*** | .41*** | .29*** | .14*** | .08** | .25*** | .33*** | .24*** | .32*** | .20*** | .29*** | |  |
| Item 5 |  |  |  |  | - | .44*** | .31*** | .43*** | .39*** | .27*** | .31*** | .27*** | .39*** | .33*** | .50*** | .33*** | .59*** | .14*** | .12*** | .36*** | .29*** | .12*** | .41*** | .21*** | .24*** | |  |
| Item 6 |  |  |  |  |  | - | .21*** | .29*** | .25*** | .27*** | .21*** | .22*** | .25*** | .20*** | .33*** | .22*** | .36*** | .16*** | .15*** | .34*** | .21*** | .10*** | .36*** | .19*** | .20*** | |  |
| Item 7 |  |  |  |  |  |  | - | .31*** | .25*** | .47*** | .39*** | .40*** | .39*** | .53*** | .47*** | .31*** | .24*** | .14*** | .20*** | .15*** | .63*** | .18*** | .30*** | .18*** | .36*** | |  |
| Item 8 |  |  |  |  |  |  |  | - | .48*** | .26*** | .43*** | .26*** | .31*** | .31*** | .43*** | .53*** | .38*** | .08** | .06** | .24*** | .31*** | .22*** | .37*** | .23*** | .26*** | |  |
| Item 9 |  |  |  |  |  |  |  |  | - | .24*** | .46*** | .19*** | .25*** | .24*** | .34*** | .37*** | .39*** | .13*** | .08** | .23*** | .23*** | .12*** | .32*** | .25*** | .16*** | |  |
| Item 10 |  |  |  |  |  |  |  |  |  | - | .27*** | .36*** | .37*** | .41*** | .38*** | .26*** | .21*** | .13*** | .28*** | .24*** | .45*** | .16*** | .27*** | .16*** | .32*** | |  |
| Item 11 |  |  |  |  |  |  |  |  |  |  | - | .28*** | .41*** | .36*** | .46*** | .45*** | .23*** | .11*** | .12*** | .16*** | .38*** | .22*** | .31*** | .19*** | .26*** | |  |
| Item 12 |  |  |  |  |  |  |  |  |  |  |  | - | .40*** | .38*** | .34*** | .25*** | .19*** | .05* | .20*** | .20*** | .39*** | .23*** | .25*** | .17*** | .59*** | |  |
| Item 13 |  |  |  |  |  |  |  |  |  |  |  |  | - | .37*** | .45*** | .32*** | .27*** | .11*** | .25*** | .17*** | .42*** | .16*** | .33*** | .16*** | .32*** | |  |
| Item 14 |  |  |  |  |  |  |  |  |  |  |  |  |  | - | .49*** | .31*** | .24*** | .15*** | .19*** | .21*** | .59*** | .17*** | .28*** | .25*** | .30*** | |  |
| Item 15 |  |  |  |  |  |  |  |  |  |  |  |  |  |  | - | .44*** | .40*** | .14*** | .13*** | .25*** | .46*** | .16*** | .44*** | .22*** | .27*** | |  |
| Item 16 |  |  |  |  |  |  |  |  |  |  |  |  |  |  |  | - | .26*** | .12*** | .09*** | .14*** | .33*** | .22*** | .31*** | .13*** | .26*** | |  |
| Item 17 |  |  |  |  |  |  |  |  |  |  |  |  |  |  |  |  | - | .09*** | .10*** | .36*** | .24*** | .08** | .42*** | .22*** | .16*** | |  |
| Item 18 |  |  |  |  |  |  |  |  |  |  |  |  |  |  |  |  |  | - | .10*** | .10*** | .12*** | .07** | .10*** | .17*** | .07** | |  |
| Item 19 |  |  |  |  |  |  |  |  |  |  |  |  |  |  |  |  |  |  | - | .22*** | .26*** | .15*** | .12*** | .06* | .20*** | |  |
| Item 20 |  |  |  |  |  |  |  |  |  |  |  |  |  |  |  |  |  |  |  | - | .20*** | .16*** | .30*** | .17*** | .17** | |  |
| Item 21 |  |  |  |  |  |  |  |  |  |  |  |  |  |  |  |  |  |  |  |  | - | .19*** | .30*** | .23*** | .36*** | |  |
| Item 22 |  |  |  |  |  |  |  |  |  |  |  |  |  |  |  |  |  |  |  |  |  | - | .24*** | .11*** | .28*** | |  |
| Item 23 |  |  |  |  |  |  |  |  |  |  |  |  |  |  |  |  |  |  |  |  |  |  | - | .17*** | .26*** | |  |
| Item 24 |  |  |  |  |  |  |  |  |  |  |  |  |  |  |  |  |  |  |  |  |  |  |  | - | .17*** | |  |
| Item 25 |  |  |  |  |  |  |  |  |  |  |  |  |  |  |  |  |  |  |  |  |  |  |  |  | - | |  |
| *Note*. ^***^p < .01; ^**^p < .01; ^*^p < .05. (2-tailed), *n* = 1,750 | | | | | | | | | | | | | | | | | | | | | | | | | | | |
