# Supplementary material for: Psychometric properties of the Maternal Postnatal Attachment Scale and the Postpartum Bonding Questionnaire in three German samples
Source: BMC Pregnancy Childbirth. 2024 Nov 26;24:789. doi: 10.1186/s12884-024-06964-4 (PMC11590467; doi:10.1186/s12884-024-06964-4)
Supplement: Supplementary file 4 — Supplementary Material 4 [file 12884_2024_6964_MOESM4_ESM.docx]

**Supplement 4**

*Scree plots and tests of MPAS and PBQ in the three analyzed data sets*

| Non-graphical solutions to scree test MPAS_PP,7M_**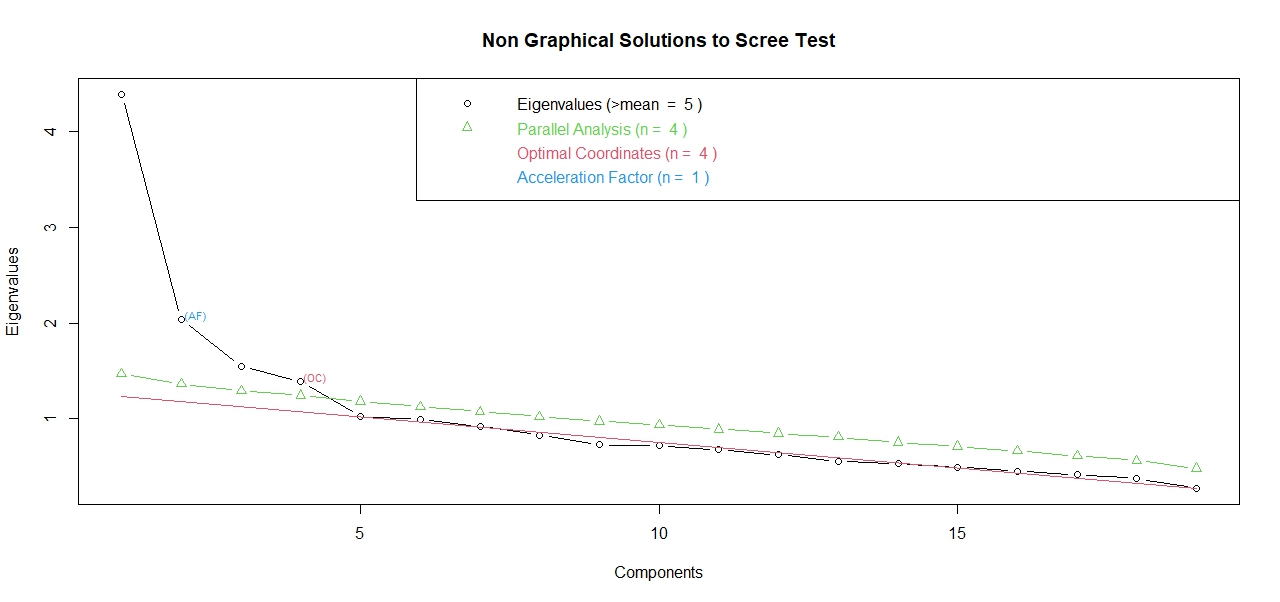** |
| --- |
| Non-graphical solutions to scree test MPAS_PP,12M_**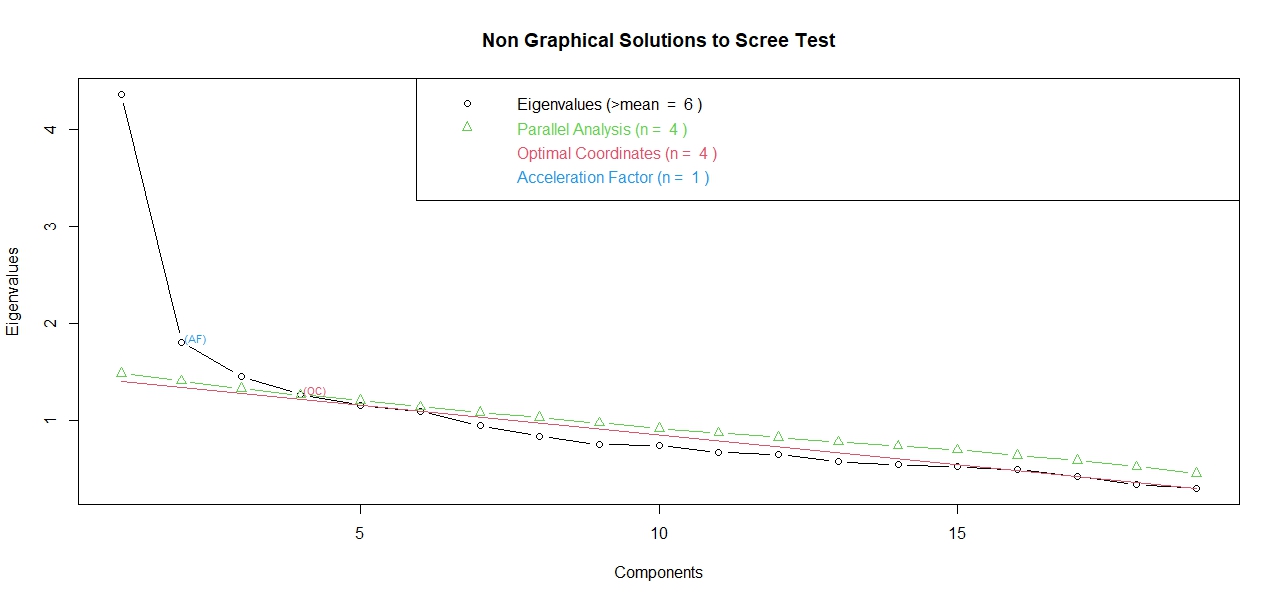** |
| Non-graphical solutions to scree test MPAS_M,4M_**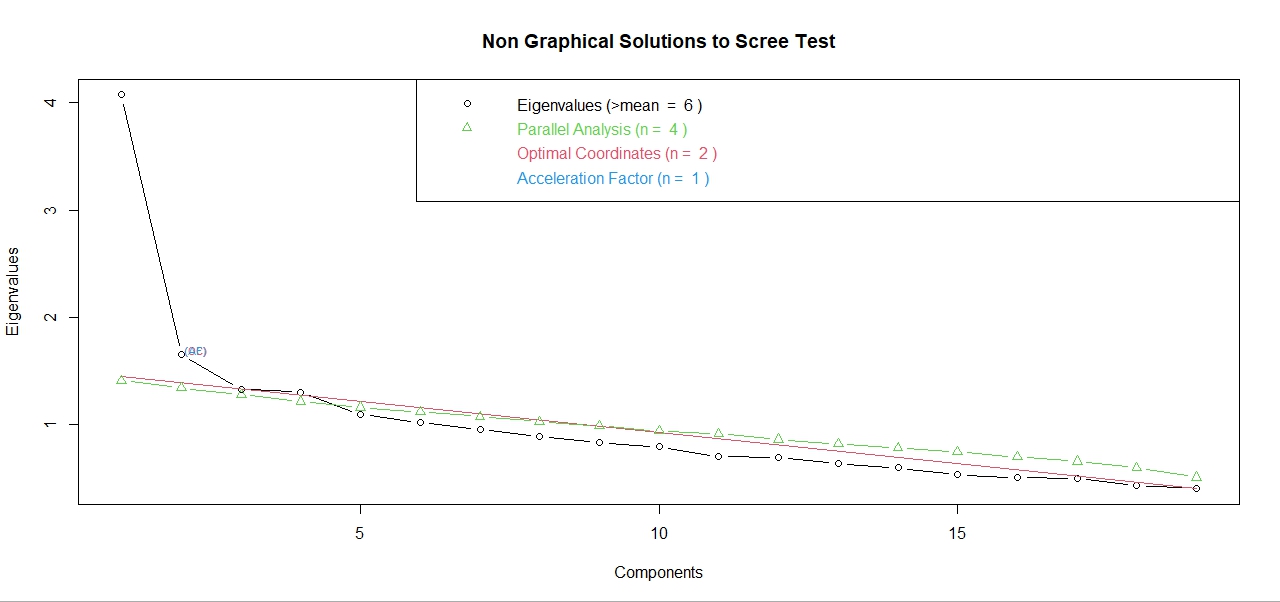** |

*Note:* Acceleration Factor (AF) marking where the slope of the curve changes abruptly.

| Non-graphical solutions to scree test PBQ_M,2M_  **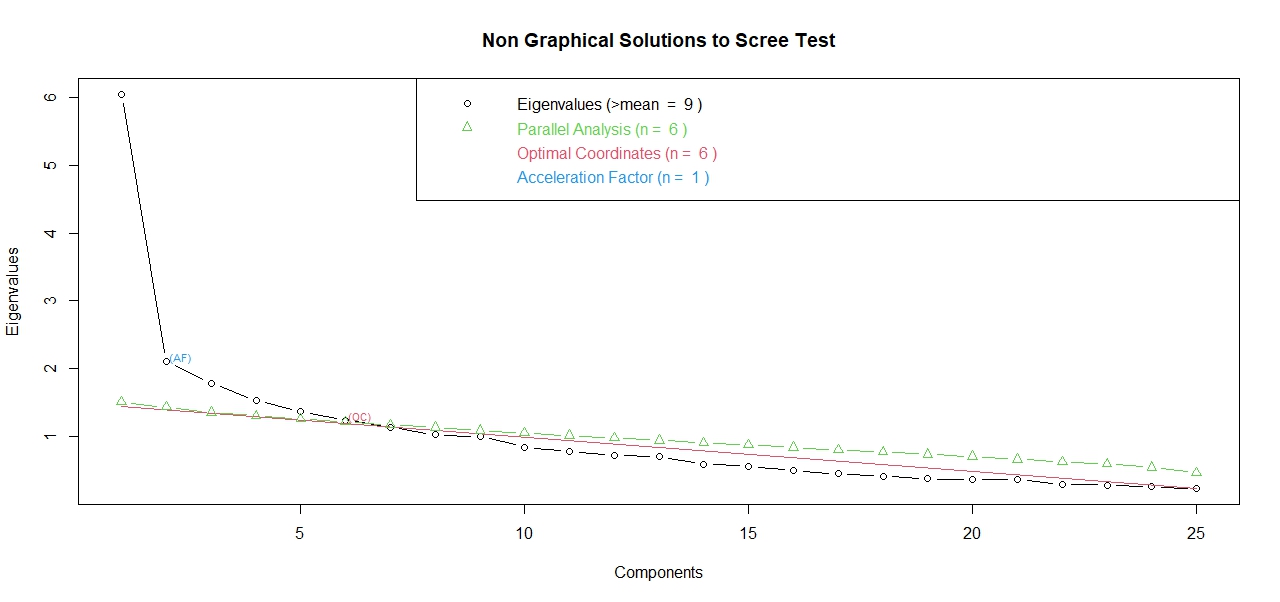** |
| --- |
| Non-graphical solutions to scree test PBQ_D,2M_  **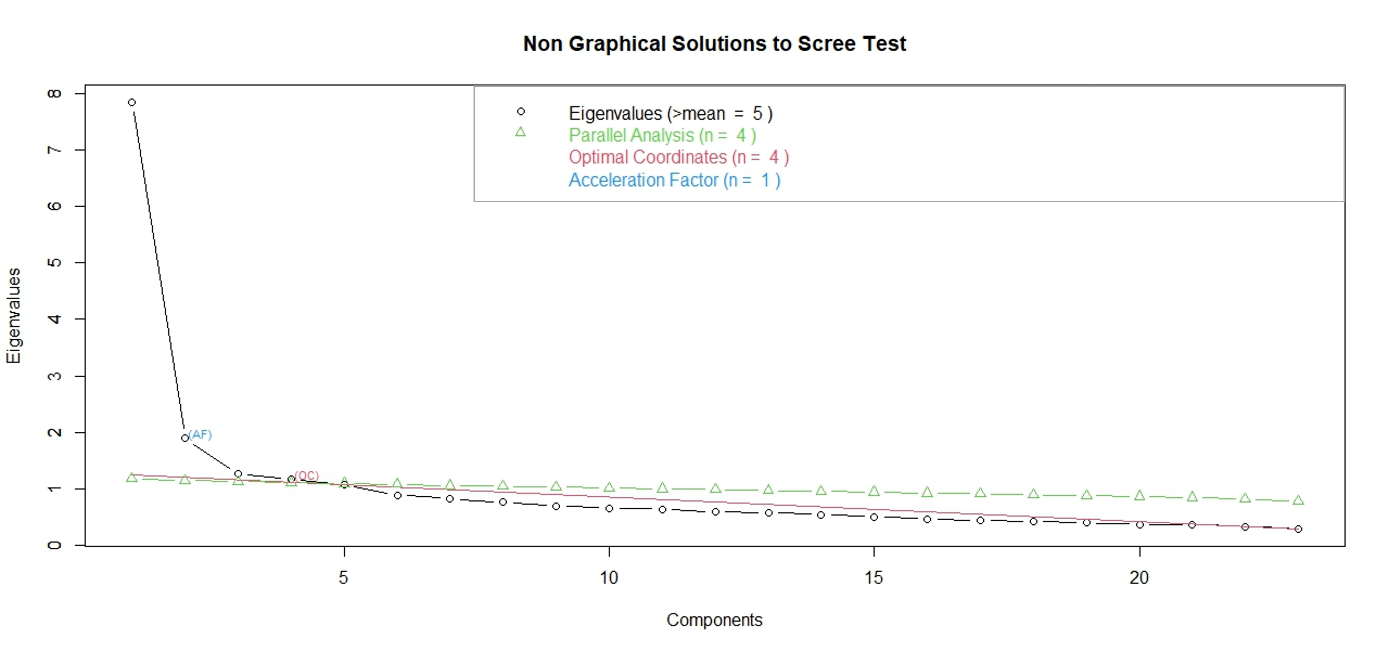** |
| Non-graphical solutions to scree test PBQ_D,14M_  **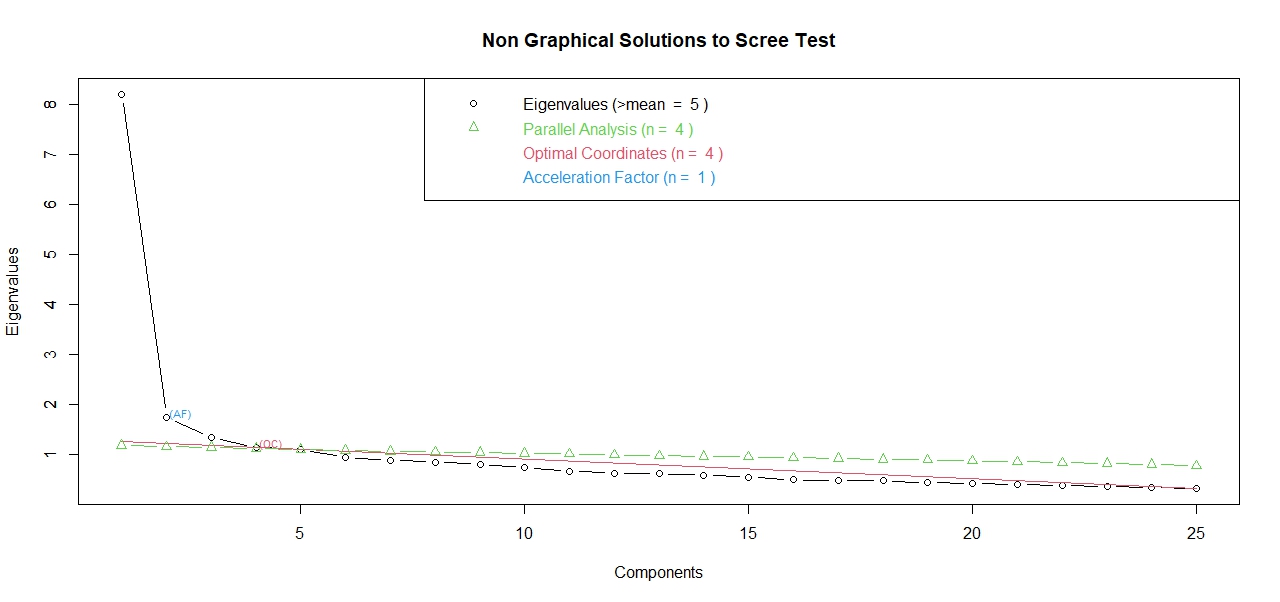** |

*Note:* Acceleration Factor (AF) marking where the slope of the curve changes abruptly.
