# Supplementary material for: Psychometric properties of the Maternal Postnatal Attachment Scale and the Postpartum Bonding Questionnaire in three German samples
Source: BMC Pregnancy Childbirth. 2024 Nov 26;24:789. doi: 10.1186/s12884-024-06964-4 (PMC11590467; doi:10.1186/s12884-024-06964-4)
Supplement: Supplementary file 5 — Supplementary Material 5 [file 12884_2024_6964_MOESM5_ESM.docx]

| **Supplement 5**  MPAS One-factor solution | | | | | | | | |
| --- | --- | --- | --- | --- | --- | --- | --- | --- |
|  | |  | MPAS_PP,7M_ (*n*=218) | | MPAS_PP,12M_ (*n*=197) | | MPAS_M,4M_ (*n*=281) | |
| Item (original factor) | |  | F1 | *h*^2^ | F1 | *h*^2^ | F1 | *h*^2^ |
| 1 | Annoyance | (AH) | 0.71 | 0.51 | 0.55 | 0.32 | 0.61 | 0.37 |
| 2 | Difficult child | (AH) | 0.40 | 0.16 | 0.31 | 0.10 | 0.37 | 0.13 |
| 3 | Affection | (QA) | 0.46 | 0.21 | 0.54 | 0.29 | 0.24 | 0.06 |
| 4 | Guilt | (QA) | 0.46 | 0.21 | 0.37 | 0.14 | 0.45 | 0.20 |
| 5 | Competence | (QA) | 0.45 | 0.20 | 0.53 | 0.29 | 0.35 | 0.13 |
| 6 | Tension | (QA) | 0.45 | 0.20 | 0.42 | 0.18 | 0.57 | 0.32 |
| 8 | Play | (PI) | *0.28* | *0.08* | *0.27* | *0.07* | *0.28* | *0.08* |
| 9 | Separation | (PI) | *0.19* | *0.04* | *0.22* | *0.05* | 0.34 | 0.11 |
| 10 | Enjoyment | (QA) | 0.61 | 0.38 | 0.61 | 0.37 | 0.50 | 0.25 |
| 11 | Thoughts on baby | (PI) | 0.33 | .0.11 | 0.31 | 0.10 | *0.15* | *0.02* |
| 12 | Prolong/reduce time | (PI) | 0.51 | 0.31 | 0.57 | 0.32 | 0.34 | 0.11 |
| 13 | Meet again | (PI) | 0.44 | 0.20 | 0.49 | 0.24 | 0.43 | 0.19 |
| 14 | Own Baby | (QA) | 0.30 | 0.93 | 0.55 | 0.30 | *0.19* | *0.04* |
| 15 | Resent Baby | (AH) | 0.46 | 0.21 | 0.44 | 0.19 | 0.49 | 0.24 |
| 16 | Time for oneself | (AH) | 0.43 | 0.19 | 0.49 | 0.24 | 0.44 | 0.19 |
| 17 | Burden | (AH) | 0.33 | 0.11 | 0.39 | 0.15 | 0.58 | 0.34 |
| 18 | Trust in own judgement | (QA) | *0.27* | *0.07* | 0.34 | 0.12 | 0.42 | 0.17 |
| 19 | Impatience | (QA) | 0.47 | 0.22 | 0.33 | 0.11 | 0.41 | 0.17 |
| Eigenvalue | |  | 3.49 |  | 3.58 |  | 3.21 |  |
| Variance, % | |  | 0.19 |  | 0.20 |  | 0.17 |  |
| *Note.* In brackets MPAS original factors; Values in grey and italics mark factor loadings <.30; Item 7 as excluded from this analysis beforehand due to low factor loadings; QA, quality of attachment; PI, pleasure in interaction; AH, absence of hostility; *h*^2^, communalities | | | | | | | | |
