# Supplementary material for: Psychometric properties of the Maternal Postnatal Attachment Scale and the Postpartum Bonding Questionnaire in three German samples
Source: BMC Pregnancy Childbirth. 2024 Nov 26;24:789. doi: 10.1186/s12884-024-06964-4 (PMC11590467; doi:10.1186/s12884-024-06964-4)
Supplement: Supplementary file 6 — Supplementary Material 6 [file 12884_2024_6964_MOESM6_ESM.docx]

| **Supplement 6**  PBQ One-factor solution | | | | | | | | | | |
| --- | --- | --- | --- | --- | --- | --- | --- | --- | --- | --- |
|  | |  | PBQ_M,2M_ (*n*=281) | | PBQ_D,2M_ (*n*=1,840) | | | PBQ_D,14M_ (*n*=1,750) | | |
| Item (original factor) | |  | F1 | *h^2^* | | F1 | *h^2^* | | F1 | *h^2^* |
| 1 | Close to baby | (IB) | 0.66 | 0.43 | | 0.70 | 0.50 | | 0.68 | 0.46 |
| 2 | Wish old days back | (IB) | 0.56 | 0.32 | | 0.65 | 0.42 | | 0.56 | 0.32 |
| 3 | Distant from baby | (RA) | 0.50 | 0.25 | | 0.70 | 0.49 | | 0.71 | 0.50 |
| 4 | Love to cuddle | (RA) | 0.47 | 0.22 | | 0.62 | 0.38 | | 0.59 | 0.34 |
| 5 | Regret having baby | (RA) | 0.34 | 0.12 | | 0.55 | 0.31 | | 0.63 | 0.40 |
| 6 | Baby not mine | (IB) | *0.18* | *0.03* | | 0.49 | 0.24 | | 0.48 | 0.24 |
| 7 | Baby winds up | (IB) | 0.61 | 0.37 | | 0.60 | 0.37 | | 0.63 | 0.40 |
| 8 | Love for baby | (IB) | 0.45 | 0.20 | | 0.69 | 0.47 | | 0.62 | 0.38 |
| 9 | Feel happy w baby | (IB) | 0.36 | 0.13 | | 0.49 | 0.24 | | 0.51 | 0.26 |
| 10 | Baby irritates | (IB) | 0.58 | 0.33 | | 0.58 | 0.33 | | 0.56 | 0.31 |
| 11 | Enjoy playing | (RA) | 0.61 | 0.36 | | 0.62 | 0.39 | | 0.62 | 0.38 |
| 12 | Baby crying | (IB) | 0.35 | 0.12 | | 0.44 | 0.20 | | 0.55 | 0.30 |
| 13 | Trapped as mother | (IB) | 0.60 | .35 | | 0.58 | 0.33 | | 0.60 | 0.36 |
| 14 | Angry with baby | (RA) | 0.56 | 0.32 | | 0.55 | 0.30 | | 0.61 | 0.37 |
| 15 | Resent baby | (IB) | 0.64 | 0.41 | | 0.65 | 0.42 | | 0.70 | 0.49 |
| 16 | Baby most beautiful | (IB) | 0.49 | 0.24 | | 0.56 | 0.32 | | 0.56 | 0.31 |
| 17 | Wish baby go away | (IB) | 0.31 | 0.09 | | 0.56 | 0.32 | | 0.51 | 0.26 |
| 19 | Baby makes anxious | (AC) | 0.37 | 0.14 | | 0.31 | 0.10 | | *0.28* | *0.08* |
| 20 | Afraid of baby | (AC) | 0.34 | 0.12 | | 0.39 | 0.15 | | 0.40 | 0.16 |
| 21 | Baby annoys | (RA) | 0.65 | 0.43 | | 0.62 | 0.39 | | 0.64 | 0.42 |
| 22 | Feeling confident | (AC) | *0.18* | *0.03* | | 0.36 | 0.13 | | 0.32 | 0.10 |
| 23 | Someone else look  after baby | (RA) | 0.28 | 0.08 | | 0.52 | 0.28 | | 0.56 | 0.32 |
| 24 | Hurting baby | (AB) | *0.10* | *0.01* | | *0.16* | *0.03* | | 0.33 | 0.11 |
| 25 | Baby easily comforted | (AC) | 0.41 | 0.17 | | 0.45 | 0.20 | | 0.50 | 0.26 |
|  | Eigenvalue |  | 5.28 |  | | 7.28 |  | | 7.51 |  |
|  | Variance, % |  | 0.22 |  | | 0.30 |  | | 0.31 |  |
| *Note*. In brackets PBQ original factors; Values in grey and italics mark factor loadings <.30; Item 18 was excluded from this analysis beforehand due to low factor loadings; IB, impaired bonding; RA, rejection and anger; AC, anxiety about care; AB, risk of abuse, *h^2^*, communalities | | | | | | | | | | |
